# Implications of inflammation and sex in lower extremity arterial disease

**DOI:** 10.1111/eci.70144

**Published:** 2025-11-05

**Authors:** Katja Schnidrig, Manovriti Thakur, Aleksandra Tuleja, Sarah Maike Bernhard, Heidi Noels, Drosos Kotelis, Marc Schindewolf, Yvonne Döring

**Affiliations:** ^1^ Division of Angiology, Swiss Cardiovascular Center Inselspital, Bern University Hospital, University of Bern Bern Switzerland; ^2^ Department for BioMedical Research (DBMR) University of Bern Bern Switzerland; ^3^ Graduate School for Health Sciences University of Bern Bern Switzerland; ^4^ Institute for Molecular Cardiovascular Research (IMCAR) RWTH Aachen University Aachen Germany; ^5^ Department of Biochemistry, CARIM, Cardiovascular Research Institute Maastricht Maastricht University Maastricht the Netherlands; ^6^ Aachen‐Maastricht Institute for Cardiorenal Disease (AMICARE) University Hospital RWTH Aachen Aachen Germany; ^7^ Department of Vascular Surgery Bern University Hospital, University of Bern Bern Switzerland; ^8^ Institute for Cardiovascular Prevention (IPEK) Ludwig‐Maximilians‐University Munich (LMU) Munich Germany; ^9^ DZHK (German Centre for Cardiovascular Research) Partner Site Munich Heart Alliance Munich Germany

**Keywords:** atherosclerosis, inflammation, LEAD, sex differences

## Abstract

**Background:**

Lower extremity arterial disease (LEAD) affects over 200 million people globally and is largely driven by chronic vascular inflammation. However, the complex interplay between inflammatory pathways, their prognostic value and potential sex‐specific differences remains insufficiently understood.

**Methods and Results:**

Literature indicates that elevated inflammatory markers—such as (high‐sensitivity) C‐reactive protein, fibrinogen, D‐dimer, interleukin‐6, α‐defensins and soluble adhesion molecules as well as newly arising parameters such as neutrophil counts and markers of clonal haematopoiesis—may predict both the onset and progression of LEAD, from declining ankle–brachial indices and impaired walking performance to higher rates of amputation, cardiovascular events and mortality. Moreover, women with LEAD frequently present at older ages with more advanced disease, exhibit distinct lesion patterns and greater functional impairment, and often have higher baseline CRP levels than men, although the strength of association between inflammatory markers and adverse outcomes may be attenuated in women. However, it remains unclear how inflammatory markers can guide (sex) specific patient stratification in LEAD or which markers provide the most clinical utility in general.

**Conclusion:**

Together, these findings underscore the need for comprehensive inflammatory profiling in LEAD risk stratification and highlight the importance of joining sex‐specific analyses, new (bio)markers and machine learning to integrate clinical, genomic, proteomic and functional data into future studies to inform patient‐tailored prevention and treatment strategies.

## Graphical abstract

The graphical abstract illustrates the role of inflammation and sex‐related differences in lower extremity artery disease (LEAD). Key inflammatory biomarkers associated with LEAD include C‐reactive protein (CRP), fibrinogen, interleukin‐6 (IL‐6), and D‐dimer, which contribute to vascular inflammation and atherosclerotic plaque formation in lower limb arteries. Sex‐specific differences are observed, with men showing higher prevalence of smoking, more typical symptoms, a stronger CRP‐LEAD association, and more frequent iliac or infragenicular lesions. Women, on the other hand, are more likely to present with hypertension, older age at diagnosis, more asymptomatic disease courses, and predominantly femoropopliteal lesions. Associations with diabetes, prevalence, outcome, and dyslipidemia remain uncertain for both sexes.

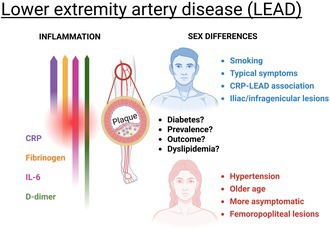

## 1 INTRODUCTION

Atherosclerotic cardiovascular disease (CVD) and its clinical complications (e.g. myocardial infarction (MI) and stroke) are the leading cause of death worldwide. It is well established that hyperlipidemia, particularly the accumulation of modified lipids (e.g. *oxidized low‐density lipoprotein* – oxLDL), initiates endothelial dysfunction. Activated endothelium, which upregulates the expression of vascular adhesion molecules, attracts leukocytes which infiltrate the tunica intima (most inner layer of arteries) thereby contributing to subintimal lesion growth. Growing lesions may cause vessel narrowing or occlusion, while plaque rupture triggers acute arterial thrombosis and its complications. The hallmark of growing lesions is infiltrating monocytes which differentiate into macrophages and engulf lipids. Lipid overload transforms these cells into foam cells that eventually undergo apoptosis and/or necrosis, thereby participating in the development of a necrotic core. Furthermore, vascular smooth muscle cells (VSMCs), populating the tunica media (second arterial layer), undergo a phenotypic switch from a contractile to a synthetic state which enables them to migrate into the intima. Intimal VSMCs accumulate underneath the plaque cap where they produce extracellular matrix to stabilize the lesion, which further triggers intimal thickening. In general, atherosclerotic plaques can be divided into stable and unstable lesions. Stable plaques are characterized by a dense and thick fibrous cap, whereas unstable lesions contain a large lipid core and a thin fibrous cap rendering them more prone to rupture.^1^, ^2^, ^3^

Atherosclerosis affects all vascular territories, yet non‐coronary manifestations—particularly those involving the lower extremities—remain understudied and suffer from suboptimal treatment. Adding to the challenge is the inconsistent terminology: historically, the terms *peripheral artery or arterial disease* (PAD) or *peripheral vascular disease* have been poorly defined and often used to encompass a wide range of vascular disorders excluding coronary artery disease, including carotid, vertebral, upper extremity, renal, mesenteric and lower extremity arteries.^4^ The 2017 Guidelines from the European Society of Cardiology (ESC) and the European Society for Vascular Surgery (ESVS) clarified this by defining *peripheral arterial diseases* as all arterial diseases excluding the coronary arteries and the aorta, while reserving *peripheral artery disease* more specifically for lower extremity artery disease (LEAD).^5^ Hence, in this review, we will focus exclusively on the lower extremities and therefore use the term LEAD. Given that LEAD shares inflammatory processes with related chronic conditions—including CVD, type 2 diabetes (DM2), metabolic syndrome and obesity, as well as chronic kidney disease (CKD)^6^, ^7^, ^8^, ^9^, ^10^—we consider directing on inflammation as a unifying mechanism to be both timely and well supported by recent literature. In addition, we will examine how sex differences influence disease presentation, progression and outcomes.

## 2 LEAD

More than 200 million people suffer from LEAD worldwide. LEAD incidence starts rising from around 50 years and increases sharply after the age of 65 years.^11^ The clinical spectrum ranges from patients who are asymptomatic, to intermittent claudication (IC) (leg pain triggered by exercise, relieved by rest) up to critical limb ischemia (CLI) including rest pain, wounds/ulcers and gangrene. The classification is usually made by the Fontaine or Rutherford classification, and both are focused on symptom severity (Table 1).

**TABLE 1:** Fontaine and Rutherford classification of LEAD according to Aboyans et al.^5^

Fontaine		Rutherford	
Category	Symptoms	Category	Symptoms
I	Asymptomatic	0	Asymptomatic
II		1	Mild intermittent claudication
IIa	Intermittent claudication >200 m	2	Moderate intermittent claudication
IIb	Intermittent claudication <200 m	3	Severe intermittent claudication
III	Rest pain	4	Rest pain
IV	Ulceration or gangrene	5	Minor tissue loss
		6	Ulceration or gangrene

The prevalence of LEAD in the population is estimated around 3%–10%,^5^ but varying definitions in the different studies focusing on IC, ankle brachial index (ABI) or other criteria complicate exact statistics. In a systematic review in 2013, the prevalence was studied with the definition of an ABI ≤.9 comparing populations in high‐income countries (HIC) and low‐income and middle‐income countries (LMIC). The prevalence of LEAD rises steadily with age—from about 5% among 45–49‐year‐olds to roughly 18% in the 85–89 age group—highlighting its strong age dependency. Projections over the next decade estimate a 28.7% increase in cases in low‐ and middle‐income countries and a 13.1% rise in high‐income countries, driven largely by increased life expectancy and underscoring a growing global burden.^11^

Although atherosclerosis is a systemic disease with shared risk factors across its major manifestations (coronary, cerebral, and peripheral), patients with LEAD appear to have a disproportionately high risk of cardiovascular events. In the REACH registry in 2007, Steg et al. showed that the incidences of cardiovascular death, MI, stroke or hospitalization for atherothrombotic events were 15.2% for coronary artery disease (CAD) patients, 14.5% for cerebrovascular disease patients and 21.1% for LEAD patients.^12^ In addition, Sabouret et al. demonstrated that comorbidity across vascular territories is common: approximately one quarter of patients with CAD, one third of those with cerebrovascular disease, and half of LEAD patients also had atherothrombotic disease in at least one other vascular region.^13^ Moreover, a meta‐analysis revealed a cumulative incidence of 13% in symptomatic LEAD patients in comparison to 5% in the reference population (without LEAD).^14^ These three studies highlight that LEAD patients are frequently affected by polyvascular disease. More importantly, they appear to carry the highest cardiovascular risk burden compared to those with coronary or cerebrovascular disease, emphasizing the urgent need to intensify LEAD‐specific research and clinical focus.

### 2.1 Risk factors

Risk factors for LEAD are the traditional cardiovascular risk factors (smoking, diabetes, dyslipidemia and hypertension), but their impact on LEAD differs in comparison to other atherosclerotic diseases and vascular beds. For example, although smoking is one of the strongest risk factors for atherosclerosis in general, it seems to be a particularly important risk factor for LEAD. Smokers have a two to four times higher risk of developing LEAD.^5^ In addition, the association between smoking and LEAD seems to significantly diminish after 10 years of cessation, but after 20 years an increased residual risk still remains.^15^

Another strong correlation has been observed between the duration of diabetes mellitus (DM) and the risk of developing LEAD, with longer disease duration significantly increasing the likelihood of peripheral arterial involvement.^5^ Importantly, disease prognosis in diabetic LEAD patients is significantly worse than in non‐diabetic patients, with a five times higher risk for amputations and a significant increase in mortality.^16^ Moreover, in diabetic LEAD patients vascular complications are more frequent in arteries distal to the knee compared to patients without diabetes and partly explain why those patients have a higher risk for amputations.^16^

Hypertension and high cholesterol have also been associated with LEAD but they seem to be of lower significance as risk factors than smoking and DM.^17^

Beyond traditional factors, various non‐traditional risk factors including ethnicity, alcohol consumption, hyperhomocysteinemia, CKD, sex and gender, obesity, poverty, inflammation, as well as genetic predisposition have been investigated and shown to be associations with LEAD.^10^, ^17^, ^18^ For instance, a genome‐wide association study (GWAS) identified nineteen LEAD‐associated loci, of which eleven shared a disease association in three vascular beds (cerebral, coronary and peripheral (LEAD)). Single nucleotide polymorphism (SNP) rs7903146 within TCF7L2, as a known genetic factor for DM2, was associated with LEAD and rs7903146 was significantly attenuated when controlled for DM2, indicating that this SNP impacts the LEAD risk through its effect on DM2. Further, this study identified four LEAD‐specific SNPs, one being F5 p.R506Q, a Factor V Leiden variant (thrombophilia), and CHRNA3, which was previously shown to predict nicotine addiction. The other two (HLA‐B, RP11‐359 M6.3) are novel and their potential mechanistic implications remain elusive. In conclusion, the authors suggested that smoking and thrombosis may play an even greater role in LEAD than in any other vascular territory.^19^

Another emerging risk factor is clonal haematopoiesis (CH), characterized by acquired mutations in haematopoietic stem cells leading to mutant clone expansion. CH increases the risk of haematological cancer and non‐haematological diseases, particularly atherosclerosis and cardiovascular disease. Whether CH is a cause or a consequence of atherosclerosis remains controversial. However, a recent study using high‐sensitivity DNA sequencing and vascular imaging showed that CH mutations raise the risk of femoral atherosclerosis over 6 years, but atherosclerosis itself does not influence mutant cell growth. These findings suggest that CH particularly promotes lower extremity atherosclerosis, thereby highlighting its potential as a target for cardiovascular disease prevention.^20^ Another example of a non‐traditional risk factor is inflammation, for which an association with LEAD has been shown.^17^ In the following paragraphs we will further explore the implications of inflammation in the context of LEAD.

## 3 INFLAMMATION AND LEAD

It is now widely accepted that atherosclerosis results from the complex interplay between classical risk factors—such as DM2, smoking, hypertension and hypercholesterolemia—and associated endothelial dysfunction and chronic vascular inflammation.^21^, ^22^ Here we focus on the role of inflammation, particularly in LEAD.

### 3.1 Inflammatory markers as biomarkers for LEAD development

Already in 1998, a prospective case–control study by Ridker et al. showed in 144 healthy men with a follow‐up of 60 months an association between c‐reactive protein (CRP) levels and the development of LEAD. Significantly higher CRP levels at baseline were found among those who developed LEAD during the follow‐up. Moreover, the risk of developing LEAD increased with increasing quartile of CRP level and stayed similar after correcting for confounding factors.^23^ These results were confirmed in a big case–control study (mean follow‐up 9 years) with 14,916 initially healthy men. This study measured 11 lipid and non‐lipid biomarkers (e.g. fibrinogen, CRP, homocysteine) at baseline and found that among the non‐lipid biomarkers, men who developed LEAD had significantly higher levels of fibrinogen and CRP, with CRP emerging as the strongest predictor based on multivariable analyses.^24^ Similarly, the Atherosclerosis Risk in Communities (ARIC) study published in 2020 with 9851 subjects free of LEAD at baseline and a mean follow‐up of 17.4 years demonstrated an association of CRP with the incidence of LEAD, independent of traditional risk factors. In addition, galectin 3, a marker that has been linked to atherogenesis and vascular remodelling (the expression can be upregulated in vascular smooth muscle cells and contributes to vascular fibrosis) was monitored and was associated with the development of LEAD independently of traditional risk factors and CRP. Notably, the two markers together modestly improved the prediction of LEAD.^25^ The Edinburgh Artery cohort study (2007) with a follow‐up of 17 years and a population of 1519 subjects (51% males) without LEAD at recruitment, examined 17 potential blood markers (inflammatory, hemostatic and rheological markers) as possible predictors of LEAD. After adjustment for cardiovascular risk factors and baseline CVD, only CRP and fibrinogen showed a significant association with LEAD development. Risk association for other markers like interleukin (IL)‐6, soluble intercellular adhesion molecule‐1 (sICAM‐1) and d‐dimer was attenuated when adjusted for cardiovascular risk factors, whereas soluble vascular adhesion molecule‐1 (sVCAM‐1) showed no association at all with the development of LEAD. The authors concluded that their findings demonstrate independent associations of inflammatory markers with LEAD incidence (even independent of traditional risk factors).^26^ Another prospective case–control study with 14,916 middle‐aged men focused on sICAM‐1 and sVCAM‐1. Development of symptomatic LEAD was monitored during a 9‐year follow‐up and revealed elevated levels of sICAM‐1 but not of sVCAM‐1 to be associated with the subsequent development of LEAD. In contrast to the Edinburgh Artery Study, this association remained after adjustment for possible confounding factors and elevated sICAM‐1 stayed significant after adjustment for CRP. Hence, a combination of sICAM‐1 and CRP seemed to identify male subjects at the greatest risk of suffering from LEAD.^27^

Others have investigated neutrophil counts in the context of cardiovascular disease. They conducted observational studies involving 101,730 subjects (45% male) and genetic studies using one‐sample Mendelian randomization (365,913 subjects, 46.3% male) and two‐sample Mendelian randomization (563,085 subjects). These analyses revealed that higher neutrophil counts are causally associated with an increased risk of LEAD. While several previous studies have demonstrated an association between neutrophil counts and cardiovascular disease risk, this study now provides strong evidence for a causal role for neutrophils in the development of LEAD and potentially other cardiovascular diseases.^28^ Taken together, multiple large‐scale prospective studies (Table 2) have demonstrated a consistent and independent association between elevated inflammatory markers—particularly CRP, fibrinogen, sICAM‐1, galectin‐3 and neutrophil counts—and the future development of LEAD. These findings not only support inflammation as a key driver of LEAD pathogenesis but also suggest that certain biomarkers, alone or in combination, may improve risk prediction beyond traditional cardiovascular risk factors. However, although CRP and IL‐6 have been linked to LEAD development, no universally accepted thresholds currently exist to guide clinical decision making and further prospective studies are needed to define high‐risk populations.

**TABLE 2:** Longitudinal Studies – Inflammatory markers in LEAD.

Study population#	% male/female	Follow up (years)	Markers sig. associated with LEAD	References
Inflammatory markers and LEAD development				
144, healthy	100/0	5y	CRP base. ↑	Ridker 1998^23^
14,916, healthy	100/0	Mean 9y	CRP and fibrinogen base. ↑	Ridker 2001^24^
14,916, healthy	100/0	Mean 9y	sICAM1 ↑	Pradhan 2002^27^
1519, healthy	51/49	17y	CRP, fibrinogen, LP(a), haematocrit ↑	Tzoulaki 2007^26^
9851, healthy	44/66	Median 17.4y	hs‐CRP, galectin 3 ↑	Ding 2020^25^
101,730, healthy^a^	45/55	Median 9.5y	Neutrophil counts ↑	Luo 2023^28^
Inflammatory markers and LEAD progression				
1081, healthy	51/49	5y	IL‐6 ↑ assoc. with ABI change	Tzoulaki 2005^30^
813, healthy	51/49	12y	CRP, IL‐6, ICAM1 ↑ assoc. with ABI change	Tzoulaki 2005^30^
387 LEAD pat.	68/32	Median 2y	hs‐CRP ↑ assoc. with LEAD severity and ABI ↓	Vainas 2005^31^
403 LEAD pat.	87/13	Mean 4.6y ±2.5y	hs‐CRP ↑ assoc. with ABI ↓	Aboyans 2006^32^
332 (270 LEAD pat.)	58/42	Mean 3y	hs‐CRP, d‐Dimer ↑ but no assoc. with LEAD progr.	Musicant 2006^33^
487 (296 LEAD pat.)	60/40	Mean 3y	hs‐CRP ↑ assoc. with decline in 6 min walk perform.	McDermott 2006^34^
Inflammatory markers and association to CVD events in LEAD				
486 LEAD pat.	61/39	Median 7y	Fibrinogen ↑ assoc. with all cause death	Doweik 2003^44^
387 LEAD pat.	68/32	Median 2y	hs‐CRP ↑ assoc. with CVD events/death	Vainas 2005^31^
75 LEAD pat.	79/21	Median 2y	sVCAM ↑ assoc. with CVD events	Silvestro 2005^48^
452 LEAD pat.	58/42	Mean 2.1y ±1.4y	hs‐CRP ↑ assoc. with CVD events/death/amputation	Hogh 2008^38^
377 LEAD pat.	100/0	Mean 3.4y	hs‐CRP, d‐Dimer ↑ assoc. with all cause death in first 2y	Vidula 2008^39^
156 LEAD pat.	77/23	Median 17.5y	hs‐CRP no assoc. with MI or stroke	Brevetti 2008^41^
785 LEAD pat.	100/0	Median 4.6y	Fibrinogen ↑ assoc. with all cause death	Bartlett 2009^45^
397 LEAD pat.	88/12	Mean 7y	hs‐CRP ↑ assoc. with all cause death only after 2y	Criqui 2010^40^
463 LEAD pat.	58/42	Mean 6.1y	hs‐CRP ↑ assoc. with 5x higher CVD events/CVD death	Urbonaviciene 2012^35^
595 LEAD pat.	64/36	Mean 3y	d‐Dimer ↑ assoc. with IHD within first 2 month only	McDermott 2016^42^
1363 LEAD pat.	86/14	Mean 1.5y	Fibrinogen ↑ assoc. with MI, stroke, amputation & bleeding	Altes 2018^46^
254 LEAD pat.	71/29	Unclear	IL‐6 ↑ assoc. with amputation/all cause death	Gremmels 2019^37^
335 LEAD pat.	82/18	Median 3.6y	hs‐CRP ↑ assoc. with CVD and all cause death	Fukase 2021^36^
5041 LEAD pat.	Mixed	Meta‐analysis	hs‐CRP ↑ assoc. with CVD events	Singh 2017^43^
21,473 LEAD pat.	Mixed	Meta‐analysis	hs‐CRP, fibrinogen, d‐dimer, NT‐proBNP, hs‐cTnT ↑ assoc. with CVD events/death and all cause death	Kremers 2020^47^
Inflammatory markers and association to LEAD severity/clinic				
1156, healthy	51/49	5y	hs‐CRP ↑ assoc. with LEAD severity at baseline	Tzoulaki 2005^30^
463 LEAD pat.	58/42	Mean 6.1y	hs‐CRP ↑ assoc. with CLI compared IC	Urbonaviciene 2012^35^
255 LEAD pat.	59/41	Mean 3y	IL‐6 ↑ assoc. functional decline	McDermott 2011^55^

^a^ In addition: One‐sample Mendelian randomization showed higher neutrophil counts = higher risk for LEAD (odds ratio 1.19).

Further, while CRP and IL‐6 are frequently elevated in patients with LEAD, one should keep in mind that they represent markers of generalized systemic inflammation. Moreover, femoral artery lesions—common in LEAD—are histologically characterized by high degrees of fibrosis and calcification but lower macrophage burden and pro‐inflammatory cytokine activity compared to coronary or carotid plaques.^29^ Hence, we have to carefully discriminate between systemic inflammation in LEAD patients which may reflect multisite atherosclerotic disease activity and comorbidities versus markers which may mechanistically be associated with LEAD in particular. Hence, while CRP and IL‐6 may be useful for risk stratification, future studies are needed incorporating vascular‐bed‐specific biomarker signatures, ideally with also spatial resolution to compare disease mechanisms on a molecular level.

### 3.2 Inflammatory markers and LEAD progression

In the Edinburgh Artery Study 1.592 subjects (51% male) were assessed for five inflammatory markers—CRP, IL‐6, sICAM‐1, sVCAM‐1 and E‐selectin—as potential predictors of LEAD progression over a 12‐year follow‐up. ABI measurements were taken at three time points: baseline, 5 years and 12 years. The inflammatory markers were measured only at baseline, and analyses were adjusted for baseline risk factors. IL‐6 was associated with LEAD progression, indicated by a lower ABI compared to baseline, at both 5 and 12 years. In contrast, CRP and sICAM‐1 showed an association with progression only at the 12‐year mark.^30^ This suggests that CRP, IL‐6 and sICAM‐1 are all markers associated with progression but IL‐6 is the most consistent and strongest predictive marker for LEAD in this setting. Associations for sVCAM‐1 and ABI change were not significant.^30^ Another study measured CRP levels in 387 patients with LEAD (68.2% male) and examined their relationship with ABI at baseline and after 12 months of follow‐up. Patients were divided into three groups based on baseline CRP levels. ABI at baseline showed a significant decline from the low‐CRP to the high‐CRP group over the 12‐month period, even after adjusting for conventional risk factors.^31^ In a cohort of 403 subjects (87% male), Aboyans et al.^32^ demonstrated that after adjusting for traditional risk factors, CRP was an independent and significant predictor of LEAD progression. Both ABI and toe‐brachial index (TBI) were measured at baseline and after a mean follow‐up of 4.6 ± 2.5 years. The study was based on the hypothesis that risk factors influencing large‐vessel disease (assessed by ABI) may differ from those affecting small‐vessel disease (assessed by TBI). Major progression was defined as being in the highest decile of decline: a decrease of ≥.3 in ABI or ≥.27 in TBI. During follow‐up, 10.6% of patients exhibited a major decrease in ABI, and 7.1% showed a major decrease in TBI. Multivariable analysis revealed that current smoking, changes in lipid profile, and elevated CRP levels were associated with large‐vessel LEAD progression, whereas only diabetes was significantly associated with small‐vessel LEAD progression. The authors concluded that inflammation, smoking, and dyslipidemia contribute to large‐vessel disease progression, while diabetes is the primary driver of small‐vessel involvement.^32^ In contrast to the previously mentioned findings, a prospective study involving 332 subjects (57.8% male) with a mean follow‐up of 38.4 months found no association between CRP levels and LEAD progression. In this study, CRP and d‐dimer were measured at baseline, and clinical follow‐up assessments, including ABI measurements, were conducted every 6 months. The authors suggested that the lack of association may be due to limitations such as the relatively small sample size and the relatively short follow‐up duration. They also questioned the utility of non‐specific inflammatory markers like CRP in predicting LEAD progression, highlighting the need for more precise biomarkers.^33^ Nevertheless, a study of 296 patients (59.8% male) evaluated CRP and d‐dimer levels at baseline and annually over a three‐year period, alongside assessments of physical function including the 6‐min walk test, a rapid‐paced 4‐m walk, and a summary performance score. The findings showed that elevated CRP levels were associated with a greater annual decline in 6‐min walk performance in patients with LEAD. In contrast, d‐dimer levels were not linked to functional decline.^34^ In summary, multiple studies have identified CRP, IL‐6 and sICAM‐1 as inflammatory markers associated with LEAD progression (Tables 2 and 3), with IL‐6 emerging as the most consistent predictor. While some evidence also supports CRP levels reflecting vascular and functional decline, other studies have questioned its utility, emphasizing the need for more specific biomarkers.

**TABLE 3:** Cross‐sectional Studies – Inflammatory markers in LEAD.

Study population #	% male/female	Markers sig. associated with LEAD	References
Inflammatory markers and association to LEAD severity/clinic			
1823 healthy	48/52	CRP ↑ assoc. with lower ABI in men only	Folsom 2001^49^
601 (of whom 370 LEAD pat.)	58/42	CRP, d‐dimer ↑ assoc. functional decline	McDermott 2003^52^
202 (of whom 162 LEAD pat.)	69/31	CRP, d‐dimer, vWVF ↑ assoc. with CLI & IC	Cassar 2005^50^
127 LEAD pat.	64/36	IL‐6 assoc. invers. with max tredmill walk. distance	Nylænde 2006^54^
423 LEAD pat.	54/46	IL‐6, d‐dimer, sICAM1, sVCAM ↑ assoc. with functional decline	McDermott 2008^53^
126 pat. (of whom 51 LEAD and 75 With no history of PAD or CVD)	59/41	sVCAM ↑ not assoc. with LEAD severity	Edlinger 2019^51^
Inflammatory markers and association to prevalent LEAD			
955 (of whom 105 LEAD pat.)	52/48	CRP, IL‐6, d‐dimer ↑ assoc. with prevalent LEAD	McDermott 2005^56^
4787 (of whom 5% LEAD pat.)	51/49	CRP, fibrinogen, leukocyte # ↑ assoc. with prevalent LEAD	Wildman 2005^57^
2800 (of whom 111 LEAD pat.)	47/53	CRP, IL‐6 fibrinogen, TNF‐⍺, TNFR‐2: assoc. invers. to ABI	Murabito 2009^58^
878, unclear	100/0	IL‐6, TNF‐⍺ ↑ assoc. with prevalent LEAD	Cauley 2016^59^
152 (of whom 80 LEAD pat.)	Unclear	IL‐6, TNF‐⍺, sVCAM, sICAM1 ↑ assoc. with prevalent LEAD	Signorelli 2016^60^
126 pat. (of whom 51 LEAD and 75 With no history of PAD or CVD)	59/41	sVCAM ↑ assoc. with prevalent LEAD	Edlinger 2019^51^

### 3.3 Inflammatory markers and their association with cardiovascular events in LEAD

As previously noted, patients with LEAD face a high cardiovascular risk—even greater than that of patients with CAD. Moreover, this elevated risk in LEAD appears to be less dependent on traditional risk factors and several studies support the notion that inflammation may serve as an independent risk factor for future cardiovascular events in LEAD patients.

Vainas et al. investigated the association between CRP levels and future cardiovascular events in patients with LEAD. Over a median follow‐up of 24 months, they found that higher CRP levels were associated with an increased risk of a composite endpoint of death and/or any cardiovascular event. The majority of events were peripheral in nature, characterized by worsening ischemic symptoms alongside a significant decrease in ABI or the need for peripheral revascularization procedures.^31^ Urbonaviciene et al. confirmed this relation between CRP and cardiovascular event rate. They measured plasma α‐defensin (an antimicrobial peptide of the innate immune system, sequestered in the granules of neutrophils) and CRP in 463 LEAD patients (58% male) and examined their relationship with cardiovascular mortality. Patients with IC and high α‐defensin and CRP concentrations had a five times higher risk for cardiovascular mortality than those with either only one parameter being increased or low levels of both markers.^35^ When combined with traditional risk factors, CRP levels significantly improved the accuracy of cardiovascular mortality risk prediction. However, this association was not observed in patients with critical limb ischemia. In this subgroup, although higher CRP levels were initially linked to an increased risk of cardiovascular death, the association lost statistical significance after adjusting for age, sex and other cardiovascular risk factors.^35^ A study involving 335 patients with intermittent claudication (82% male) examined the prognostic impact of CRP levels and found that both all‐cause mortality and cardiovascular‐related death were significantly higher in the high‐CRP group compared to the low‐CRP group. However, there was no significant difference in the cumulative incidence of major adverse cardiovascular limb events between the two groups.^36^ With respect to IL‐6, a study with 254 LEAD patients (all with severe limb ischemia, 71.3% male) found significantly elevated levels in patients with major events (amputation or death).^37^ An association between CRP levels and future events was also observed in a study of 452 LEAD patients (57.5% male), where CRP was measured at baseline and patients were followed for a mean of 2.1 years. Higher CRP levels were found in those who died, required amputation, or experienced lower limb thrombosis. Yet, the authors emphasized that CRP is a nonspecific marker influenced by various conditions and therefore should not be used as a standalone predictor.^38^

Vidula et al. observed time‐dependent associations between biomarkers and mortality in 377 LEAD patients (61.8% male). D‐dimer, CRP and serum amyloid A were significantly associated with cardiovascular and all‐cause mortality within 2 years of initial measurement; however, their prognostic value diminished for deaths occurring between years two and three.^39^ Similarly, in a cohort study of 397 patients, CRP was a significant predictor of mortality at two‐year follow‐up, but this association was no longer evident over the full follow‐up period (mean 7 years).^40^ Other markers included in this study (serum amyloid A, d‐dimer, platelet‐activating factor, homocysteine, lipoprotein a) did not show any association.^40^ Taken together, the authors concluded that CRP may be a strong predictor of short‐term mortality in LEAD patients and may help identify individuals who require aggressive risk factor modification.^40^ Yet, the predictive value of CRP was questioned in another small cohort study of 156 LEAD patients (76.9% male), where no significant association was found between CRP levels and the risk of future MI or stroke over a median follow‐up of 17.5 months—even after adjusting for traditional risk factors and potential confounders.^41^ Similar findings were reported in the Biomarker Risk Assessment in Vulnerable Outpatients (BRAVO) study, which followed 595 LEAD patients (64% male) over 3 years, collecting blood samples every 2 months to assess CRP, d‐dimer and serum amyloid A. The primary endpoint was MI, unstable angina or death from ischemic heart disease. D‐dimer levels were significantly elevated within 2 months of an event compared to levels 10–32 months prior. Notably, median d‐dimer levels were already elevated in cases versus controls as early as eight and 4 months before the event. In contrast, CRP and serum amyloid A did not consistently rise prior to events, suggesting limited predictive value.^42^ While CRP appears to be associated with cardiovascular events in LEAD patients, the evidence remains inconsistent. Recently, Singh et al. conducted a systematic review and meta‐analysis to evaluate this relationship and allow for more robust conclusions. Focusing on major cardiovascular outcomes including myocardial infarction, stroke, cardiac revascularization and mortality, their review included 16 studies with a total of 5041 participants, of which 8 studies were eligible for meta‐analysis. Overall, patients with elevated CRP levels had a significantly higher risk of cardiovascular events compared to those with lower levels. The authors concluded that elevated CRP may indicate increased cardiovascular risk in LEAD patients, but they still point out the urgent need for larger randomized studies to also determine whether targeting inflammation could effectively reduce events in this high‐risk subgroup.^43^

Fibrinogen, another marker for inflammation (acute‐phase protein) and coagulation, was investigated in 486 LEAD patients (61% male) with a median follow‐up of 7 years for all‐cause and cardiovascular mortality. Fibrinogen was measured at baseline and divided into quartiles. Patients in the third and fourth quartiles (highest fibrinogen levels) had a significant increase in all‐cause mortality compared to patients in the lowest quartile.^44^ These results were confirmed in 2009 in a study involving 785 men, where fibrinogen was independently associated with mortality risk in LEAD, namely at 6 months and 3 years of follow up.^45^ Similarly, Altes et al. measured fibrinogen levels at baseline in 1363 LEAD patients (86.2% male) and reported elevated fibrinogen levels at baseline that were associated with an over 2‐fold higher rate of ischemic stroke, limb amputation or death and an even over 3‐fold higher rate of major bleeding.^46^ A meta‐analysis of 47 studies involving 21,473 LEAD patients evaluated several biomarkers, including CRP, fibrinogen, d‐dimer, N‐terminal pro‐B‐type natriuretic peptide, high‐sensitivity cardiac troponin T and adiponectin. Among inflammatory markers, elevated CRP levels were associated with a relative risk of 1.86 (95% CI: 1.48–2.33) for major cardiovascular events and 3.49 (95% CI: 2.35–5.19) for mortality. Fibrinogen and d‐dimer were also linked to increased mortality risk, with relative risks of 2.08 (1.46–2.97) and 2.22 (1.24–3.98), respectively. The authors concluded that CRP, fibrinogen and d‐dimer are promising biomarkers for identifying LEAD patients at heightened risk of adverse cardiovascular outcomes.^47^ In addition, adhesion molecules may also hold prognostic value for cardiovascular events. In a small study of 75 LEAD patients, elevated plasma levels of sVCAM‐1 were associated with a 3.4‐fold increased risk of cardiovascular events, independent of traditional risk factors.^48^ Hence, inflammatory biomarkers—especially CRP, fibrinogen and d‐dimer—may help identify those LEAD patients at greater risk for adverse events (Tables 2 and 3). Nevertheless, while CRP shows a consistent association with short‐term outcomes, its long‐term predictive value remains uncertain.

In summary, given the mixed prognostic value of inflammatory markers, routine measurement of CRP or IL‐6 cannot yet be recommended for clinical decision‐making in LEAD. However, short‐term elevations of these markers—particularly within the first 30 days—may still help flag high‐risk individuals in select cases, such as those with critical limb ischemia. Meta‐analyses support this role in risk stratification, but results are still not robust enough and warrant further large‐scale clinical studies to determine whether targeting inflammation can improve outcomes in LEAD.

### 3.4 Inflammatory markers and their association with LEAD severity and clinical function

IC results from flow‐limiting stenoses or occlusions, leading to impaired blood flow distal to the lesion. Some studies suggest that inflammation may contribute to reduced walking capacity and may also serve as a marker of LEAD severity, as reflected by changes in ABI or clinical staging systems such as Fontaine or Rutherford (Table 1).

As early as 2001, a large cross‐sectional study by Folsom et al. (1823 participants, 45% male) found a weak inverse association between CRP levels and ABI in men, independent of other risk factors; however, no such association was observed in women.^49^ In contrast, Urbonaviciene et al. reported significantly higher CRP levels in patients with CLI (Fontaine stage III/IV) compared to those with IC (Fontaine stage II), in both men and women.^35^ Cassar et al. found that CRP levels increased significantly with LEAD severity, with higher concentrations observed in patients with CLI compared to those with IC and healthy controls. A similar pattern was seen for d‐dimer, with levels rising in parallel with disease progression.^50^ Data for sVCAM‐1 and sICAM‐1 only showed a weak trend with LEAD severity, which was attenuated after adjustment for baseline risk factors.^30^ Furthermore, Edlinger et al. did not find any relationship between sVCAM‐1 and increasing disease stage according to Rutherford classifications. However, the small sample size and heterogeneous sex distribution (51 LEAD patients [82% male], 75 controls [35% male]) limit the strength of this finding.^51^

Focusing on functional performance, McDermott et al. reported that in 370 LEAD patients (62.2% male), but not in controls (*n* = 231, 53.2% male), higher CRP levels were associated with poorer 6‐min walk performance. No such association was observed for fibrinogen. Interestingly, higher d‐dimer levels were inversely associated with functional capacity in both LEAD patients and controls.^52^ The same group confirmed the association for higher CRP levels and poorer walk performance a few years later in a group of 423 LEAD patients (54% male), even after adjustment for confounders. Similarly, higher levels of d‐dimer, IL‐6, sVCAM‐1 and sICAM‐1 were observed in association with poorer performance.^53^

Nevertheless, another smaller study (127 LEAD patients, 64% male) did not find a significant association between function (here maximum treadmill walking distance) and CRP or sVCAM‐1 levels. IL‐6 was inversely correlated with the function independent of risk factors, and sICAM‐1 lost significance after adjustment for related risk factors.^54^ Similarly, a study of 255 LEAD patients (55.3% male) found no significant association between sVCAM‐1 levels and functional decline. However, patients with consistently high IL‐6 levels experienced a more rapid decline in function compared to those with low or fluctuating IL‐6 levels.^55^ Taken together CRP, IL‐6, and d‐dimer are associated with both disease severity and functional impairment in LEAD, including poorer walking performance and more advanced clinical stages (Tables 2 and 3). While findings for adhesion molecules like sVCAM‐1 and sICAM‐1 are less consistent, IL‐6 emerged as the most reliable indicator of functional decline across multiple studies.

### 3.5 Inflammatory markers and their association with LEAD prevalence

The inCHIANTI study, involving 955 men and women with and without LEAD, found that participants with LEAD had significantly higher levels of CRP, IL‐6, fibrinogen and IL‐1 receptor antagonist compared to those without the disease.^56^ Similarly, the National Health and Nutrition Examination Survey (NHANES), involving 4748 participants, found that CRP, fibrinogen and leukocyte count were independently associated with the presence of LEAD.^57^ In contrast, the Framingham Offspring Study, which included 2800 participants (47% men), reported that among several measured biomarkers—including CRP, fibrinogen and sICAM‐1—only IL‐6 and tumour necrosis factor receptor 2 (TNFR‐2) were significantly associated with LEAD after adjustment for confounding factors.^58^ Again, in a large cross‐sectional analysis of 878 randomly selected men, only CRP and IL‐6 were significantly associated with prevalent LEAD. Men in the highest quartiles of these markers had greater odds of LEAD compared to those in the lowest; however, the association for CRP weakened after additional adjustment for smoking.^59^ In addition, smaller studies have measured plasma levels of several biomarkers—including IL‐6, sVCAM‐1, sICAM‐1, matrix metalloproteinase‐2 (MMP‐2) and MMP‐9—in 80 LEAD patients and 72 healthy controls, finding significantly higher levels of all these markers in the LEAD group.^60^ Similarly, a more recent retrospective study involving 126 participants reported significantly elevated sVCAM‐1 levels in patients with LEAD compared to controls.^51^ However, it has to be noted that the last two studies were performed with small population sizes, limiting the clinical relevance of the proposed findings.

**FIGURE 1:**
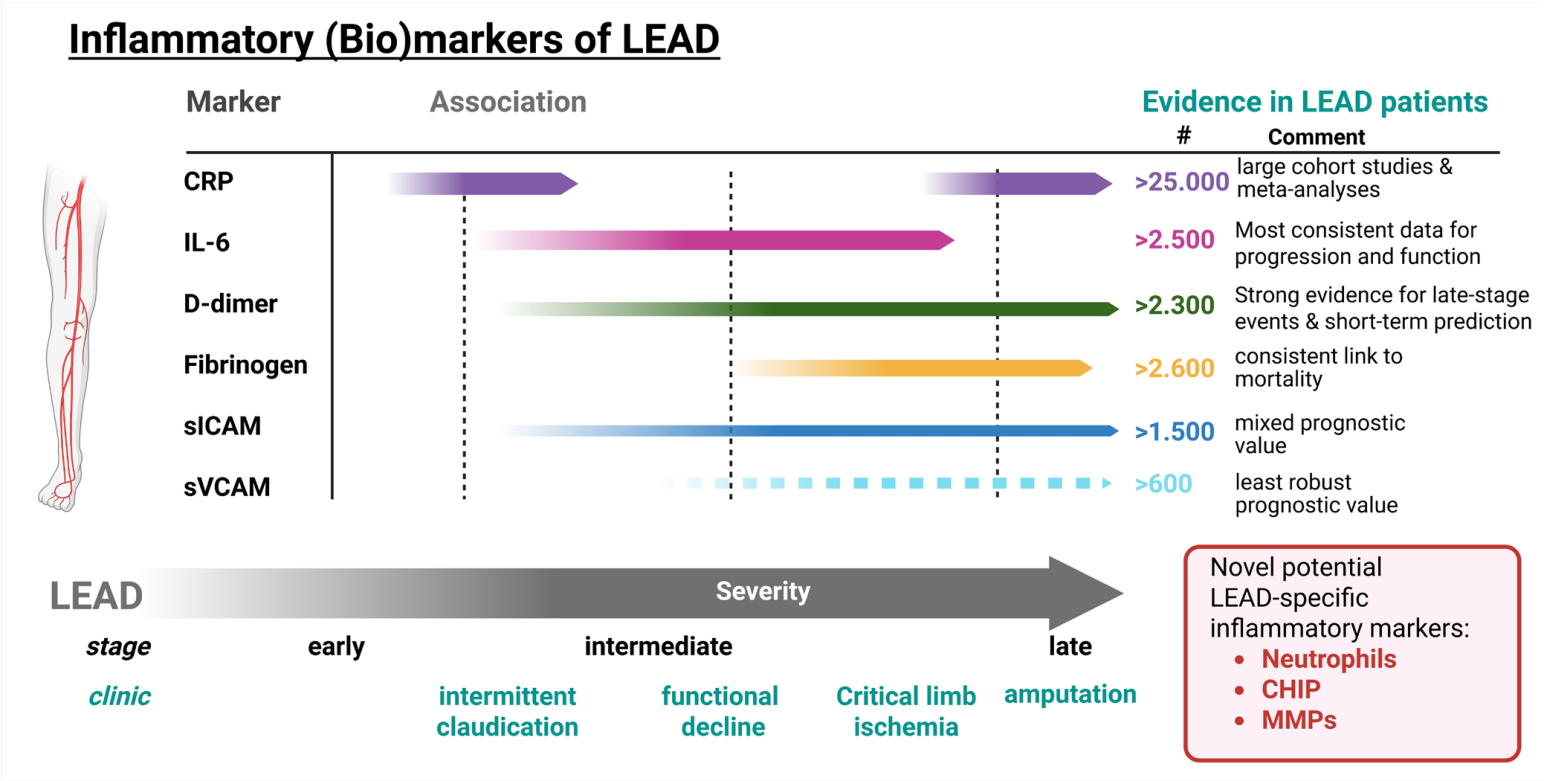
Illustration of the temporal and clinical associations of key inflammatory biomarkers with the progression of lower extremity artery disease (LEAD). Arrows represent the strength and temporal relevance of each biomarker based on published evidence in LEAD patients. Colours reflect biomarker identity, while arrow length and positioning correspond to their association with disease stages. The right panel ranks biomarkers by cumulative sample size of LEAD patients in whom associations were observed, highlighting CRP, IL‐6, d‐dimer and fibrinogen as the most studied and consistently linked markers. In contrast, sICAM and sVCAM show less robust or mixed prognostic value. A separate box highlights novel, potentially LEAD‐specific inflammatory markers—including neutrophils, clonal haematopoiesis of indeterminate potential (CHIP) and matrix metalloproteinases (MMPs)—which warrant further investigation for their mechanistic and diagnostic relevance. Some studies assessed CRP, while others used high‐sensitivity CRP (hsCRP), limiting direct comparability across datasets. *Figure was made with Biorender*.

Taken together, multiple inflammatory biomarkers have been associated with LEAD, but the most consistently reported are IL‐6 and CRP (Figure 1). However, as these markers are also elevated in other vascular conditions such as CAD, they lack specificity for LEAD, limiting their utility as LEAD‐specific diagnostic or prognostic tools. Yet, emerging evidence points now to a potential causal role of neutrophils^28^ and clonal haematopoiesis of indeterminate potential (CHIP)^20^ in the development and progression of LEAD. These novel markers are of particular interest because they may reflect underlying disease mechanisms—such as chronic inflammation and altered haematopoiesis—rather than general systemic inflammation, potentially offering greater specificity and prognostic value compared to traditional markers like CRP and IL‐6.

## 4 SEX DIFFERENCES IN LEAD

**FIGURE 2:**
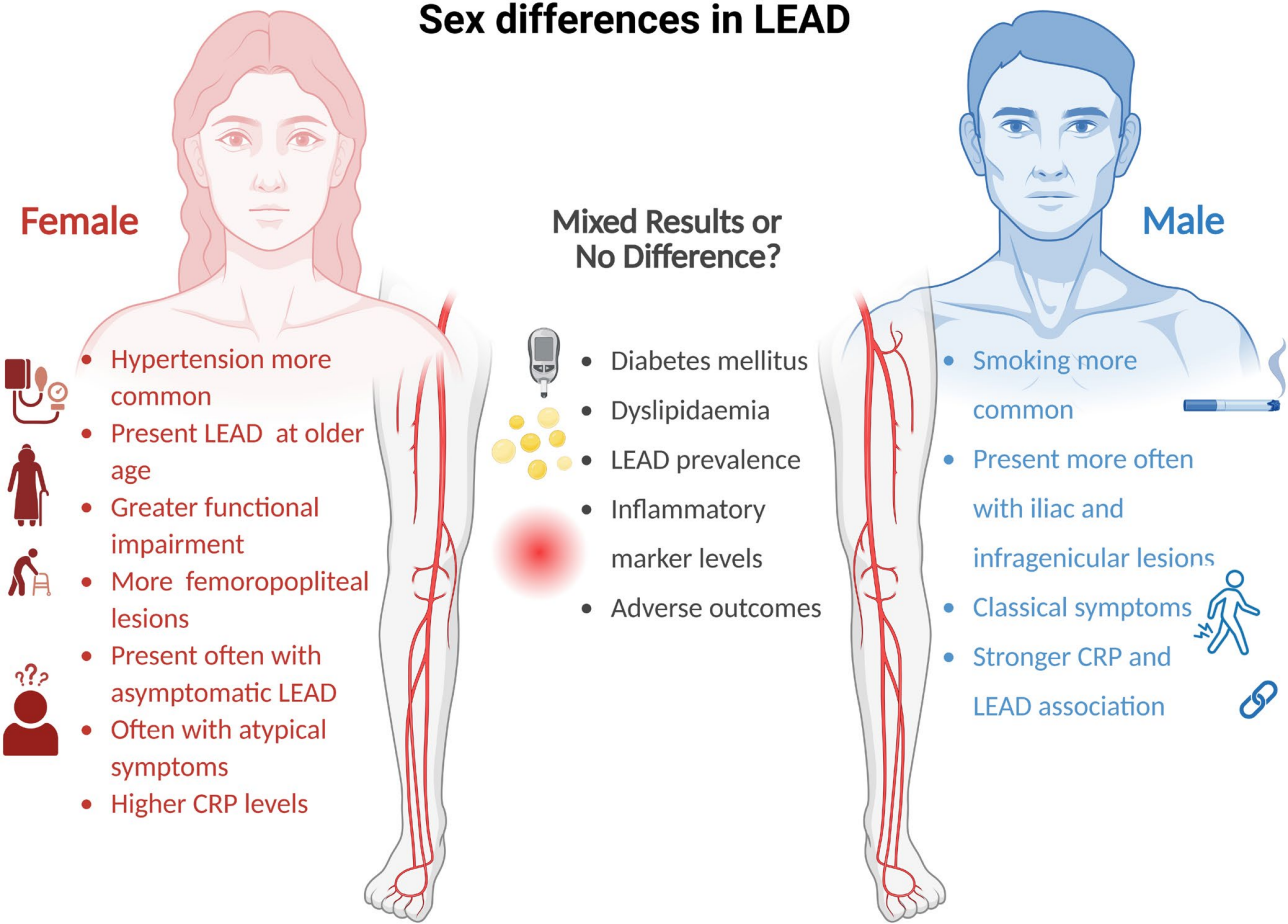
Summary of observed and reported sex differences in lower extremity artery disease (LEAD). Female patients more commonly present at an older age, often with asymptomatic or atypical symptoms, greater functional impairment, femoropopliteal lesions and higher CRP levels. In contrast, male patients more frequently smoke, show iliac and infragenicular lesion patterns, and present with classical symptoms. They also exhibit a stronger association between CRP and LEAD. The middle column highlights factors where data are mixed or inconclusive, such as diabetes, dyslipidemia, overall LEAD prevalence, inflammatory marker levels and adverse outcomes. *Figure was made with Biorender*.

Sex differences are increasingly recognized as critical in cardiovascular disease research and management (Figure 2). However, patients with LEAD remain underrepresented in cardiovascular trials compared to those with CAD or stroke, and within the limited number of LEAD‐specific studies, female participants are particularly scarce. This underrepresentation of women not only limits the generalizability of findings but also hinders our understanding of sex‐specific disease mechanisms, symptom presentation, progression, and treatment response in LEAD.^61^ Moreover, contrary to the long‐standing perception of LEAD being a predominantly male disease, growing evidence indicates that women are at least equally affected. Since LEAD prevalence increases with age—and women generally have a longer life expectancy—recognizing and understanding sex‐specific differences is essential for effective disease management, risk stratification and the development of targeted prevention strategies.^17^

### 4.1 Risk factors and sex

As previously discussed, traditional cardiovascular risk factors—such as dyslipidemia, hypertension, smoking and diabetes—play a central role in the development of LEAD and affect both sexes.^62^ The Framingham study in 1985 found DM2 to be a greater risk factor for women compared to men (4‐fold vs. 2.4‐fold increased risk) with existing impaired glucose tolerance.^63^ Similarly, another study (231 LEAD patients, 29.5% women) found DM2 more prevalent in women than in men.^64^ Furthermore, a post hoc analysis of the EUCLID trial (13,885 LEAD patients, 28% women) found LEAD women more likely to have DM2.^65^ In contrast, in a big systematic review (2,071,260 participants, 49.8% female), no evidence for a greater LEAD risk of DM (DM1 or DM2) in women compared to DM men could be found.^66^ Another analysis of 372,692 LEAD patients (43.6% female) reported lower diabetes prevalence in women than in men.^67^ This leaves us with mixed results regarding a potential sex‐dependent link between diabetes and LEAD. As mentioned earlier, smoking is an important risk factor for LEAD.^5^ Whereas Kannel et al.^63^ did not observe a sex difference in LEAD risk, other studies have reported smoking to be a stronger risk factor for LEAD in men than in women.^64^, ^67^ However, a systematic review that included 4 cohort studies (2,117,860 participants, 54.4% women) and 13 cross‐sectional studies (230,436 participants, 59.9% women) revealed differences with respect to the type of study conducted. In the cohort studies, current smoking conferred a greater excess risk of LEAD in women than in men, whereas in the cross‐sectional studies, both former and current smoking were associated with a higher LEAD risk in men.^68^ Similarly, the UK Biobank study (500,207 participants, 54.5% women) found the excess risk of LEAD greater for women than men.^69^

Regarding elevated systolic blood pressure, several studies have suggested a greater risk for women with LEAD compared to men.^65^, ^66^, ^67^ For dyslipidemia, however, the evidence is mixed. Some studies reported a higher prevalence of dyslipidemia in women with LEAD,^64^, ^65^ while others found a lower prevalence compared to men.^67^ Additionally, one study described a stronger association between high‐density lipoprotein cholesterol (HDL‐C) and LEAD specifically in women.^67^

Beyond traditional risk factors, other potential contributors to LEAD risk in women include metabolic syndrome (MetS), which is defined by a cluster of conditions such as obesity, dyslipidaemia, hypertension and glucose intolerance. MetS has been suggested as a predictor of severe LEAD. However, findings from a follow‐up study in elderly Finnish men and women indicated that this association was only present before adjusting for diabetes status. This suggests that MetS may not independently predict LEAD beyond its strong link to diabetes.^70^ In a cross‐sectional analysis of 8374 patients with DM2 (46.1% women), the presence of MetS was associated with an increased risk of LEAD in women, but not in men. This sex‐specific difference suggests that, beyond diabetes itself, other factors—such as sex hormones or sex‐related metabolic responses—may influence the relationship between MetS and LEAD, particularly in women.^71^ A prospective cohort study of 27,111 women without cardiovascular disease at baseline reported a 62% increased risk of future LEAD in those with MetS, along with a strong association between MetS and elevated levels of CRP and sICAM‐1.^72^ However, when CRP and sICAM‐1 were included in multivariable models, the association between MetS and LEAD was attenuated and no longer statistically significant. This suggests that the effect of MetS on LEAD risk may be largely mediated by inflammation and endothelial activation. Supporting sex‐specific differences in risk factors for LEAD, a systematic review and meta‐analysis found a significantly stronger association between increasing BMI and CRP levels in women compared to men.^73^ Additionally, higher CRP levels in women were also observed independent of BMI in a prospective cohort study of 6814 participants (52.8% women).^74^ These findings suggest that sex‐specific CRP levels—or inflammatory markers in general—should be considered in cardiovascular risk assessments in women.

Beyond MetS and inflammation, other potential risk factors for LEAD in women include CKD, sex hormone changes, pregnancy, hypothyroidism and osteoporosis.^75^ For instance, one study of 3174 CKD patients (45% women) found that women under 70 years of age had a 1.5‐fold higher risk for LEAD compared to men, while this sex difference disappeared in those over 70 years.^76^ This is particularly important, as further evidence (discussed below) suggests a higher LEAD prevalence in older women compared to men. Taken together, while traditional cardiovascular risk factors such as diabetes, smoking, hypertension, and dyslipidemia contribute to LEAD in both sexes, several studies suggest that their impact may differ between male and female subjects (Tables 4 and 5). Women appear to be more vulnerable to LEAD in the presence of MetS and inflammation, with elevated CRP and sICAM‐1 levels potentially mediating this risk. Additional female‐specific contributors—such as hormonal factors and conditions like osteoporosis—may further influence LEAD susceptibility, especially with increasing age.

**TABLE 4:** Longitudinal studies and meta analysis on sex differences in LEAD.

Study population #	% male/female	Follow up (years)	Factors sig. associated with sex	Sex	References
Sex and risk factors for LEAD					
5209 healthy	Unclear	26y	T2D	Women	Kannel 1985^63^
372,692 LEAD pat.	56/44	Retrospective^a^	T2D, smoking, dyslipidemia	Men	Vouyouka 2010^67^
372,692 LEAD pat.	56/44	Retrospective^a^	Hypertension	Women	Vouyouka 2010^67^
13,885 LEAD pat.	72/28	Mean 2.5y	T2D, hypertension, dyslipidemia	Women	Haine 2020^65^
500,207, mixed	45/55	Median 12.6y	Smoking	Women	Xu 2023^69^
2,071,260	50/50	Meta analysis	T2D	No diff.	Chase‐Vilchez 2020^66^
Sex and LEAD prevalence/clinic					
380 LEAD pat.	53/47	Mean 4y	Functional decline (after adj. for muscle)	No diff.	McDermott 2011^91^
60 LEAD pat.	47/53	.25y	Less functional improvement in Reha	Women	Gardner 2014^94^
24,484, mixed	Unclear	Meta analysis	Age dependent prevalence lower	Women	Hirsch 2012^79^
112,027 (of whom 9347 LEAD pat.)	Unclear	Meta analysis	LMIC: prevalence ↑, younger age	Women	Fowkes 2013^11^
Unclear	Unclear	Meta analysis	HIC: prevalence ↑	Women	Song 2019^82^
1,929,966 LEAD pat.	56/44	Meta analysis	More rest pain, atypical symptoms	Women	Porras 2022^90^
Sex and LEAD outcomes					
372,692 LEAD pat.	56/44	Retrospective^a^	Bleeding rates & mortality ↑	Women	Vouyouka 2010^67^
1,797,855 LEAD pat.	56/44	Retrospective^b^	In hospital mortality ↑	Women	Lo 2014^98^
3338 LEAD pat.	61/39	Mean 1y	Reintervention, amputation, mortality	No diff.	Ferranti 2015^99^
3338 LEAD pat.	61/39	Mean 1y	Hematoma & access site occlusions ↑	Women	Ferranti 2015^99^
10,617 LEAD pat.	49/51	Median 2.7y	Amputation, all‐cause mortality ↑	Men	Baubeta 2018^100^
13,885 LEAD pat.	72/28	Mean 2.5y	MACE, all‐cause mortality ↑	Men	Haine 2020^65^
8839 LEAD pat.	51/49	.25y	All‐cause mortality	No diff.	Mentias 2020^102^
1640 LEAD pat.	67/33	in hospital	MALE, MACE	No diff.	DeMatteis 2023^101^
94,772	56/44	Meta analysis	CVD mortality ↑	Men	Sigvant 2016^14^
2,262,387 LEAD pat.	57/43	Meta analysis	Short term adverse events a. interven. ↑	Women	Wang 2017^103^
2,262,387 LEAD pat.	57/43	Meta analysis	Long term adverse events	No diff.	Wang 2017^103^

^a^ 30 days.

^b^ In hospital.

**TABLE 5:** Cross‐sectional studies on sex differences in LEAD.

Study population #	% male/female	Factors sig. associated with sex	Sex	References
Sex and risk factors for LEAD				
231 LEAD pat.	70/30	T2D, hypertension, dyslipidemia	Women	Brevetti 2008^64^
2,117,860	46/54	Smoking (cohort study)	Women	Xu 2024^68^
230,436	40/60	Smoking (cross‐sectional)	Men	Xu 2024^68^
Sex and LEAD prevalence/clinic				
460 LEAD pat.	59/41	Stronger functional decline	Women	McDermott 2003^89^
6821 primary care pat. (among those 1230 were LEAD pat.)	42/58	<70 years higher prevalence	Men	Diehm 2004^78^
6821 primary care pat. (among those 1230 were LEAD pat.)	42/58	>85 years higher prevalence	Women	Diehm 2004^78^
2659 LEAD pat.	59/41	More often femoropopliteal lesions	Women	Diehm 2006^92^
2659 LEAD pat.	59/41	More often iliac and infragenicular lesions	Men	Diehm 2006^92^
5080 healthy (out of these 914 had LEAD)	45/55	Severe ischemia, asymptomatic, prevalence ↑	Women	Sigvant 2007^80^
231 LEAD pat.	70/30	CLI ↑	Women	Brevetti 2008^64^
231 LEAD pat.	70/30	IC ↑	Men	Brevetti 2008^64^
205,746 (population with CVD risk factors)	35/65	Prevalence ↑	Women	Hiramoto 2014^81^
233 LEAD pat.	58/42	More often femoropopliteal lesions	Women	Ortmann 2012^93^
Sex and inflammatory markers in LEAD				
1823 (541 randomly selected and 610 high risk CVD families)	48/52	Stronger inverse assoc. between CRP and ABI	Men	Folsom 2001^49^
4787 healthy (age more than 40)	51/49	CRP, fibrinogen, leukocyte counts	No diff.	Wildman 2005^57^
202 (out of this 162 LEAD pat.)	67/33	CRP	No diff.	Cassar 2005^50^
6814 CVD pat.	47/53	CRP ↑	Women	Lakoski 2006^74^
231 LEAD pat.	70/30	CRP, neutrophil & leukocyte counts	no diff.	Brevetti 2008^64^
205,746 (population with CVD risk factors)	35/65	In general higher CRP levels	Women	Hiramoto 2014^81^
205,746 (population with CVD risk factors)	35/65	Stronger association between CRP and LEAD	Men	Hiramoto 2014^81^

### 4.2 LEAD prevalence and clinical presentation differ in women and men

A large summary by Higgins et al., analysing 310 articles, reported a wide prevalence range of LEAD in women—from 3% to 29%—across ages 45–93, highlighting substantial variability in the data.^77^ Notably, two studies indicated that older women may be particularly affected by LEAD. In a cross‐sectional study of 6880 patients (58% women), women showed a lower prevalence of LEAD than men at younger ages; however, prevalence rose sharply with age and was especially high in very old women (<70 years: 11.5% in women vs. 17.1% in men; >85 years: 39.2% in women vs. 27.8% in men).^78^ Similarly, an analysis by the American Heart Association (2012) found that LEAD prevalence increased with age in both sexes. Interestingly, women aged 40–49 had a higher prevalence than men in the same age group, though prevalence tended to be lower in women at older ages. Nevertheless, the overall population burden of LEAD appeared to be greater in women.^79^

A population‐based study in Sweden involving 5080 participants (55% women) reported a higher LEAD prevalence in women (19.2%) than in men (16.5%) when defined by an ABI <.9, although the difference did not reach statistical significance.^80^ Similarly, a large screening study by Hiramoto et al., including 205,746 individuals (65% female), found a higher LEAD prevalence in women (4.1%) compared to men (2.6%).^81^ However, the study population consisted of self‐referred individuals willing to pay for screening, which introduces a potential selection bias.

In contrast, a 2013 systematic review examining populations from high‐income countries (HICs) and low‐ and middle‐income countries (LMICs) found that LEAD prevalence in HICs was similar between men and women, increasing steadily with age in both sexes (from ~5% at age 45–49 to 18% at age 85–89).^11^ In LMICs, prevalence also rose with age in both sexes, but was consistently higher in women, especially at younger ages. The authors proposed that this disparity could be due to unidentified risk factors, potential survival advantages in women or a lower baseline ABI in women compared to men.^11^ Conversely, another systematic review reported only marginally higher LEAD prevalence in women up to age 75 in HICs, and minimal, non‐significant sex differences in LMICs, conflicting with prior findings such as those by Fowkes et al.^11^, ^82^

Hence, the reported prevalence of LEAD in women is highly variable across studies. Some suggest higher rates in older women or certain age groups, while others report no significant sex differences or even lower prevalence. These inconsistencies likely reflect differences in study design, diagnostic thresholds (e.g. ABI cutoffs), population characteristics and possible sex‐specific physiological or behavioural factors.

In addition, studies directly investigating oestrogen, menopause, and hormone replacement therapy (HRT) in LEAD are lacking.^83^, ^84^, ^85^ Broader observational data suggest that postmenopausal women may experience accelerated peripheral atherosclerosis, while HRT appears to modestly lower peripheral atherosclerosis risk in some cohorts. However, major randomized trials notably Women's Health Initiative (WHI)^86^ and HERS^87^, ^88^ found no clear cardiovascular benefit and raised concerns about thrombotic complications, particularly when HRT is initiated late. These findings suggest that hormonal status may partially explain sex disparities in LEAD, but well‐designed studies specifically targeting the LEAD population are urgently needed.

Regarding symptom presentation, women with LEAD appear more likely to experience atypical symptoms and often present at more advanced disease stages compared to men. In one study, asymptomatic LEAD was significantly more common in women, and severe ischemia—defined as an ankle pressure <70 mmHg—was also more prevalent in women than men (1.5% vs. .83%).^80^ Similarly, a smaller study involving 231 participants (29.4% women) found that women were more likely to present with CLI (13.2% vs. 4.3%), while men more often had IC (89.6% vs. 77.9%).^64^ Another study of 460 LEAD patients (47.4% women) reported that compared to men, women experienced more frequently leg pain on exertion and rest —classified as atypical symptoms (27.8% vs. 13.2%).^89^ These findings are supported by a large systematic review and meta‐analysis of 1,929,966 LEAD patients (43.9% women), which showed that rest pain and atypical symptoms were more commonly reported by women (12.8% vs. 9.2%).^90^

Focusing on functional impairment, McDermott et al. found that women with LEAD exhibited more severe walking limitations than men, including slower walking speed, shorter 6‐minute walk distances and lower overall performance scores.^89^ A separate longitudinal study with annual follow‐ups over 4 years also reported greater mobility loss and a steeper functional decline in women with LEAD compared to men. Notably, these sex differences were attenuated after adjusting for baseline calf muscle area, suggesting that smaller muscle mass may partly explain the accelerated decline in women.^91^ Together, these findings indicate that women with LEAD experience greater functional impairment and a more rapid decline in mobility than men.

In addition, the localization of LEAD appears to differ by sex. In a study of 2659 patients (40.5% women) undergoing angioplasty, women were more likely to have femoropopliteal lesions, whereas men more commonly exhibited iliac and infragenicular involvement.^92^ These findings were supported by a smaller study involving 233 patients (42% women) with CLI, which reported similar sex‐specific lesion patterns.^93^ Furthermore, women—particularly those with DM2—seem to benefit less from exercise rehabilitation compared to men, as shown in a small study of 60 LEAD patients (53.4% women).^94^

Taken together, emerging evidence indicates a higher prevalence of LEAD in aging women and a greater degree of functional impairment compared to men (Tables 4 and 5). This increased decline may be attributed to both more advanced disease stages at diagnosis and lower baseline muscle mass in women. Yet, bigger and more specific cohort studies and a better understanding of sex‐related factors (including socioeconomic circumstances) are urgently needed to tailor sex and age‐specific risk profiles and subsequent therapeutic consequences.

### 4.3 Sex‐specific implications of inflammatory markers in LEAD

Not only do traditional cardiovascular risk factors differ between the sexes, but inflammatory markers also appear to show sex‐specific patterns. In the Multi‐Ethnic Study of Atherosclerosis (MESA) involving 6814 participants (52.8% women, all without known cardiovascular disease at baseline), women consistently exhibited higher CRP levels than men across all ethnic subgroups.^74^ Supporting this, a study of 687 women (30.8% with LEAD) demonstrated a clear association between elevated CRP levels and LEAD.^95^ Similarly, a large prospective study of 27,935 women without baseline vascular disease (median follow‐up: 12.3 years) found that both CRP and sICAM‐1 were significantly associated with the development of symptomatic LEAD, underscoring the role of inflammation and endothelial activation in disease onset and progression.^96^

Further highlighting sex‐specific inflammatory responses, Gardener et al. (148 LEAD patients, 51.3% female) identified differences in inflammatory and oxidative stress markers by both sex and ethnicity. Among African American participants, women had higher reactive oxygen species (ROS) production than men, suggesting greater endothelial oxidative stress. Additionally, sICAM‐1 levels were elevated in African American women, while Caucasian women had higher MMP‐9 and sVCAM‐1 levels compared to men of the same ethnicity. No significant sex differences were noted for tumour necrosis factor, IL‐6 or CRP in this cohort.^97^

Like MESA, a large voluntary screening study involving 205,746 participants (65% female) found that women—regardless of LEAD status—had higher median CRP levels than men. However, in contrast to MESA, this study reported a stronger association between CRP and LEAD (defined as ABI ≤.9) in men than in women.^81^ Supporting this, an earlier study by Folsom et al. (1823 participants, 52% female) observed a stronger inverse relationship between CRP and ABI in men compared to women.^51^ Yet, several other studies did not observe significant sex differences in inflammatory status among LEAD patients. For instance, Brevetti et al. examined 231 LEAD patients (29.5% women) and found comparable levels of CRP, leukocyte count, and neutrophil count between men and women. Yet, the low proportion of female participants may have limited the study's capacity to detect true sex‐specific effects.^64^ Nevertheless, the National Health and Nutrition Examination Survey (4787 participants, 48.9% female) found no significant sex differences in the associations between CRP, fibrinogen or leukocyte count and LEAD.^57^ Also, in a smaller study by Cassar et al. (202 participants, 33% female), CRP levels increased with LEAD severity (from controls to IC to CLI), but no sex differences were reported.^50^ In contrast, another study targeting the relationship between inflammatory markers and lethal outcomes in 463 LEAD patients (42% female), did reveal significant sex differences in CRP levels.^35^

Taken together (Tables 4 and 5), while several large studies—including MESA^74^ and a 205,746‐participant screening study^81^—consistently report higher CRP levels in women, the association between CRP and LEAD appears stronger in men in some cohorts.^49^, ^81^ Other studies, such as Gardener et al.,^97^ suggest ethnicity‐ and sex‐specific differences in inflammatory and oxidative stress markers (e.g. sICAM‐1, MMP‐9, sVCAM‐1), yet there are no consistent sex differences for CRP or IL‐6. Conversely, multiple smaller studies reported no significant sex differences in inflammatory profiles,^50^, ^57^, ^64^ while one study did find sex‐specific CRP differences linked to outcomes.^35^ These discrepancies may be due to variations in study design, sample sizes, inflammatory markers assessed and female underrepresentation in some cohorts—highlighting the need for larger, sex‐balanced studies to clarify the role of inflammation in LEAD pathophysiology.

### 4.4 Associations of sex and outcomes in LEAD

Women with LEAD have been reported to have up to a four‐fold higher risk of cardiovascular morbidity and a three‐fold increased risk of death compared to women without LEAD.^77^ Compared to men, women experienced higher mortality (5.26% vs. 4.21%, *p* < .0001) and complication rates (10.62% vs. 8.19%, *p* < .0001), as demonstrated in an analysis of 372,692 patients (43.6% female) undergoing vascular procedures (open surgery, endovascular revascularization or amputation).^67^ Similarly, in another study of 1,797,885 LEAD patients (44% female), women had higher in‐hospital mortality than men, regardless of procedure type or disease severity, even after adjusting for age and comorbidities.^98^ In contrast, Ferranti et al. studied 3338 LEAD patients (39% women) and reported similar mortality, reintervention, and major amputation rates between sexes, although women had higher rates of complications such as hematomas and access site occlusions. The study did not explore lesion characteristics but referenced other research pointing to increased bleeding risk in women.^99^ However, in a post hoc analysis of the EUCLID (Examining Use of Ticagrelor in PAD) trial, women with LEAD had even a lower risk for major adverse cardiovascular and cerebrovascular events and all‐cause mortality compared to men. Nevertheless, the EUCLID trial was not designed to evaluate sex‐related differences and the authors noted potential confounders such as selection bias, geographic, racial and ethnic variations.^65^ Yet, also a big meta‐analysis found men to have a significantly higher all‐cause mortality compared to women,^14^ and a population‐based study of 10,617 patients with CLI (50.8% female) showed men had higher rates of amputation or death following revascularization.^100^ Additionally, a retrospective study from Rome of 1640 patients (32.7% women) showed a trend toward more major adverse limb and cardiovascular/cerebrovascular events in men, though the differences were not statistically significant.^101^ Conversely, another study reported no differences between the sexes in mortality in CLI patients.^102^ A comprehensive meta‐analysis of 40 studies, however, reported that women experience worse short‐term (30‐day) outcomes, including increased risk for mortality, amputation, graft thrombosis, embolization, wound complications, cardiac events, stroke and pulmonary complications—though long‐term outcomes did not differ significantly between sexes.^103^

Taken together, while some studies suggest women with LEAD experience higher short‐term risks and complications following interventions, others report either no sex‐based differences or even better outcomes for women (Tables 4 and 5). These conflicting findings may be due to methodological differences or varying population characteristics and again underline the need for improved and careful clinical study design to better understand and address sex‐specific differences in LEAD outcomes.

## 5 THERAPEUTIC IMPLICATIONS

Despite extensive exploration of inflammatory (bio)markers such as CRP and IL‐6 in LEAD, clear therapeutic implications remain elusive. The nonspecific nature of these markers, reflecting generalized rather than site‐specific inflammation, and lack of LEAD‐specific threshold values complicate their direct translation into clinical practice. Moreover, direct comparison and subsequent conclusions on CRP levels are confounded by the fact that some studies measured CRP while others used high‐sensitivity CRP (hsCRP).

Nevertheless, also IL‐6 seems to be a strong indicator and besides the above‐mentioned clinical studies the importance of IL‐6 (at least in men) in LEAD is also supported by genetic studies. A missense variant (rs2228145; p.Asp358Ala) in IL‐6R (IL‐6 receptor) could be correlated with a higher CVD and LEAD risk as shown in data from the VA Million Veteran Program investigating blood samples of 35,042 LEAD patients and 247,115 controls (>90% male). Hence, the authors conclude that IL‐6R may represent a promising therapeutic target to reduce the risk for LEAD.^104^ Research in this direction has already been carried out in a number of other studies investigating the effect of anti‐inflammatory agents in CAD but not in LEAD.

Targeting of general inflammation in LEAD was investigated by Russel et al. who tested placebo or canakinumab treatment, a monoclonal antibody against IL‐1β, in a very small group of 38 LEAD (71% male) patients. IL‐6 and CRP fell early after treatment and they found an improvement in maximum and pain‐free walking distance when compared with placebo.^105^ However, as far as we know, there are currently no large trials which are focused on anti‐inflammatory agents in LEAD.

Additionally, emerging biomarkers, including CHIP and neutrophil subsets, hold potential but require validation in interventional studies. Ultimately, current evidence supports cautious optimism toward anti‐inflammatory therapeutic strategies but underscores a critical need for targeted clinical trials that address the complexity and heterogeneity of LEAD.

Women with LEAD often present at more advanced stages and greater walking impairment, leading to increased complications and poorer outcomes compared to men. The inconsistent findings regarding sex‐specific differences in inflammatory markers, risk‐factor severity, and clinical outcomes highlight a critical gap in current therapeutic guidelines. To improve outcomes and quality of life for female patients specifically, clinicians should proactively screen for LEAD in women, even in the absence of classic symptoms. Furthermore, future therapeutic trials and research protocols must systematically incorporate sex‐stratified analyses to enhance the accuracy of evidence‐based recommendations and treatment effectiveness for both women and men.

In summary, CRP and IL‐6 may serve as short‐term risk stratifiers but translation into clinical use will depend on whether inflammation‐targeted interventions improve outcomes in LEAD. Further, emerging markers such as CHIP and neutrophil activation may offer mechanistic specificity and warrant integration into biomarker panels and LEAD‐specific mechanistic studies.

## 6 CONCLUSION AND OUTLOOK

Despite extensive observational research, progress in understanding LEAD remains significantly limited due to a lack of sufficient mechanistic studies and subsequent clinical translation. The most problematic methodological repetition involves repeatedly using similar observational designs and nonspecific biomarkers like CRP and IL‐6. Numerous studies continue to validate associations between inflammatory biomarkers, particularly CRP and IL‐6, and LEAD incidence and progression. However, the more or less repetitive confirmation of these markers has done little to shift clinical practice, largely due to their nonspecific nature and their elevation in multiple inflammatory conditions beyond LEAD.

Notably, several meta‐analyses have also been included in this review to synthesize the available data. Yet, these too have failed to yield clear or actionable conclusions. The heterogeneity in study designs, inconsistent definitions, and variable endpoints across included studies often dilute the interpretive power of such syntheses. This highlights an important point: simply aggregating existing research may not be sufficient to clarify the complexities of LEAD. Instead, new investigative frameworks and targeted hypotheses are needed to break the current standstill.

Further, the stagnation observed in this research field may also partially be attributed to systemic factors. Biomarkers such as CRP, IL‐6 and fibrinogen are frequently chosen because of their affordability, widespread laboratory availability, and ease of measurement, leading to their repeated inclusion in studies. Additionally, publication bias toward replicating significant associations rather than exploring novel or mechanistically insightful hypotheses contributes to this dilemma. Furthermore, current funding frameworks tend to support large‐scale confirmatory observational research rather than high‐risk, innovative studies, thereby limiting the exploration of new pathways and interventions.

Simultaneously, sex differences, although acknowledged as crucial, remain inadequately addressed in most research. The still prevalent underrepresentation of women in clinical trials and the lack of sex‐specific analyses hinder the ability to develop accurate diagnostic criteria and effective treatments tailored to female patients. Women often present atypically and with more advanced disease stages, underscoring the necessity for re‐evaluation and customization of diagnostic and prognostic tools such as the ABI and inflammatory biomarker reference ranges.

To advance beyond these descriptive approaches, the LEAD field needs to shift toward integrating precision medicine approaches. This includes exploring emerging biomarkers as maybe represented by CHIP, neutrophil extracellular traps (NETs) or MMPs in combination with genomic and proteomic studies which provide new mechanistic insights. IL‐1β pathway inhibition for example demonstrated in a small‐scale intervention trial an improved functional outcome in LEAD patients receiving anti‐inflammatory therapy.^105^ Further, inclusion of machine learning to integrate clinical, genomic, proteomic, and functional data could lead to sophisticated, multi‐marker risk stratification models better suited to personalized medicine.

Dynamic profiling of biomarkers—tracking their levels and interactions over disease progression and treatment—also represents a compelling yet underexplored approach, especially in regard to post‐intervention risk stratification or treatment strategies. Unlike single, static biomarker measurements, longitudinal assessments might enhance predictive accuracy, particularly in identifying critical windows for intervention.

Ultimately, the research paradigm in LEAD requires a fundamental reorientation toward mechanistic clarity and clinical applicability. Emphasizing innovative biomarkers, integrating comprehensive phenotyping, actively addressing sex differences, and pursuing targeted (large) intervention trials will be essential steps in transforming our current observational insights into meaningful clinical advancements. Without such reorientation, LEAD research risks remaining in its descriptive capacity and staying limited in its practical impact.

## AUTHOR CONTRIBUTIONS

K.S. conducted the literature review and drafted the manuscript. M.T. and Y.D. created the figures and revised the manuscript. A.T., S.M.B., H.N., D.K., and M.S. critically contributed to manuscript writing. Y.D. performed literature screening, contributed to writing, and supervised the study.

## FUNDING INFORMATION

This research was supported by the Swiss Heart Foundation (SHF) Project ID FF20099, the Novartis foundation project number 22B148 and the Swiss National Foundation (SNF) Project ID 310030_197655 all to Y.D. This study is also funded by the German Research Foundation (DFG) Project‐ID 322900939 SFB/TRR219 (M‐05) and the European Foundation for the Study of Diabetes (EFSD)/ Boehringer Ingelheim European Research Program to H.N.

## CONFLICT OF INTEREST STATEMENT

The authors declare that the research was conducted in the absence of any commercial or financial relationships that could be construed as a potential conflict of interest.

## References

[1] Faxon DP , Fuster V , Libby P , et al. Atherosclerotic vascular disease conference. Writing group III: Pathophysiology. Circulation. 2004;109:2617‐2625. 15173044 10.1161/01.CIR.0000128520.37674.EF

[2] Libby P , Buring JE , Badimon L , et al. Atherosclerosis. Nat Rev Dis Primers. 2019;5(1):1‐18. 31420554 10.1038/s41572-019-0106-z

[3] Wang T , Butany J . Pathogenesis of atherosclerosis. Diagn Histopathol. 2017;23(11):473‐478.

[4] Criqui MH , Matsushita K , Aboyans V , et al. Lower extremity peripheral artery disease: contemporary epidemiology, management gaps, and future directions: a scientific statement from the American Heart Association. Circulation. 2021;144(9):e171‐e191. 34315230 10.1161/CIR.0000000000001005 PMC9847212

[5] Aboyans V , Ricco JB , Bartelink MLEL , et al. ESC Guidelines on the Diagnosis and Treatment of Peripheral Arterial Diseases, in collaboration with the European Society for Vascular Surgery (ESVS). Eur Heart J. 2017;39:763‐816. 10.1093/eurheartj/ehx095 28886620

[6] Ajoolabady A , Pratico D , Lin L , et al. Inflammation in atherosclerosis: pathophysiology and mechanisms. Cell Death Dis. 2024;15(11):817. 39528464 10.1038/s41419-024-07166-8 PMC11555284

[7] Hotamisligil GS . Inflammation and metabolic disorders. Nature. 2006;444(7121):860‐867. 17167474 10.1038/nature05485

[8] Sincer I , Gunes Y , Mansiroglu AK , Cosgun M , Aktas G . Association of mean platelet volume and red blood cell distribution width with coronary collateral development in stable coronary artery disease. Postepy Kardiol Interwencyjnej. 2018;14(3):263‐269. 30302102 10.5114/aic.2018.78329 PMC6173096

[9] Ziegler L , Hedin U , Gottsater A . Circulating biomarkers in lower extremity artery disease. Eur Cardiol. 2022;17:e09. 35401792 10.15420/ecr.2021.58 PMC8978021

[10] Huish S , Nawaz S , Bellasi A , Diaz‐Tocados JM , Haarhaus M , Sinha S . Clinical management of peripheral arterial disease in chronic kidney disease‐a comprehensive review from the European renal association CKD‐MBD working group. Clin Kidney J. 2025;18(5):sfaf089. 40599821 10.1093/ckj/sfaf089 PMC12209849

[11] Fowkes FGR , Rudan D , Rudan I , et al. Comparison of global estimates of prevalence and risk factors for peripheral artery disease in 2000 and 2010: a systematic review and analysis. Lancet. 2013;382(9901):1329‐1340. 23915883 10.1016/S0140-6736(13)61249-0

[12] Steg G , Bhatt DL , Wilson PWF , et al. One‐year cardiovascular event rates in outpatients with atherothrombosis. JAMA. 2007;297(11):1197‐1206. 17374814 10.1001/jama.297.11.1197

[13] Sabouret P , Cacoub P , Dallongeville J , et al. REACH: international prospective observational registry in patients at risk of atherothrombotic events. Results for the French arm at baseline and one year. Arch Cardiovasc Dis. 2008;101(2):81‐88. 18398391 10.1016/s1875-2136(08)70263-8

[14] Sigvant B , Lundin F , Wahlberg E . The risk of disease progression in peripheral arterial disease is higher than expected: a meta‐analysis of mortality and disease progression in peripheral arterial disease. Eur J Vasc Endovasc Surg. 2016;51(3):395‐403. 26777541 10.1016/j.ejvs.2015.10.022

[15] Joosten MM , Pai JK , Bertoia ML , et al. Associations between conventional cardiovascular risk factors and risk of peripheral artery disease in men. JAMA. 2012;308(16):1660‐1667. 23093164 10.1001/jama.2012.13415 PMC3733106

[16] Jude EB , Oyibo SO , Chalmers N , Boulton AJM . Peripheral arterial disease in diabetic and nondiabetic patients: a comparison of severity and outcome. Diabetes Care. 2001;24(8):1433‐1437. 11473082 10.2337/diacare.24.8.1433

[17] Criqui MH , Aboyans V . Epidemiology of peripheral artery disease. Circ Res. 2015;116(9):1509‐1526. 25908725 10.1161/CIRCRESAHA.116.303849

[18] Fowkes FGR , Aboyans V , Fowkes FJI , McDermott MM , Sampson UKA , Criqui MH . Peripheral artery disease: epidemiology and global perspectives. Nat Rev Cardiol. 2017;14(3):156‐170. 27853158 10.1038/nrcardio.2016.179

[19] Klarin D , Lynch J , Aragam K , et al. Genome‐wide association study of peripheral artery disease in the million veteran program. Nat Med. 2019;25(8):1274‐1279. 31285632 10.1038/s41591-019-0492-5 PMC6768096

[20] Díez‐Díez M , Ramos‐Neble BL , de la Barrera J , et al. Unidirectional association of clonal hematopoiesis with atherosclerosis development. Nat Med. 2024;30:2857‐2866. 39215150 10.1038/s41591-024-03213-1 PMC11485253

[21] Brevetti G , Schiano V , Chiariello M . Endothelial dysfunction: a key to the pathophysiology and natural history of peripheral arterial disease? Atherosclerosis. 2008;197(1):1‐11. 18076886 10.1016/j.atherosclerosis.2007.11.002

[22] Libby P , Ridker PM , Maseri A . Inflammation and atherosclerosis. Circulation. 2002;105(9):1135‐1143. 11877368 10.1161/hc0902.104353

[23] Ridker PM , Cushman M , Stampfer MJ , Tracy RP , Hennekens CH . Plasma concentration of C‐reactive protein and risk of developing peripheral vascular disease. Circulation. 1998;97(5):425‐428. 9490235 10.1161/01.cir.97.5.425

[24] Ridker PM , Stampfer MJ , Rifai N . Novel risk factors for systemic atherosclerosis: a comparison of C‐reactive protein, fibrinogen, homocysteine, lipoprotein(a), and standard cholesterol screening as predictors of peripheral arterial disease. JAMA. 2001;285(19):2481‐2485. 11368701 10.1001/jama.285.19.2481

[25] Ding N , Yang C , Ballew SH , et al. Fibrosis and inflammatory markers and long‐term risk of peripheral artery disease: the ARIC study. Arterioscler Thromb Vasc Biol. 2020;40(9):2322‐2331. 32698688 10.1161/ATVBAHA.120.314824 PMC7678951

[26] Tzoulaki I , Murray GD , Lee AJ , Rumley A , Lowe GDO , Fowkes FGR . Inflammatory, haemostatic, and rheological markers for incident peripheral arterial disease: Edinburgh artery study. Eur Heart J. 2007;28(3):354‐362. 17213229 10.1093/eurheartj/ehl441

[27] Pradhan AD , Rifai N , Ridker PM . Soluble intercellular adhesion molecule‐1, soluble vascular adhesion molecule‐1, and the development of symptomatic peripheral arterial disease in men. Circulation. 2002;106(7):820‐825. 12176954 10.1161/01.cir.0000025636.03561.ee

[28] Luo J , Thomassen JQ , Nordestgaard BG , Tybjærg‐Hansen A , Frikke‐Schmidt R . Neutrophil counts and cardiovascular disease. Eur Heart J. 2023;44(47):4953‐4964. 37950632 10.1093/eurheartj/ehad649 PMC10719495

[29] Soehnlein O , Lutgens E , Doring Y . Distinct inflammatory pathways shape atherosclerosis in different vascular beds. Eur Heart J. 2025;46:3261‐3272. 40036569 10.1093/eurheartj/ehaf054 PMC12401584

[30] Tzoulaki I , Murray GD , Lee AJ , Rumley A , Lowe GDO , Fowkes FGR . C‐reactive protein, interleukin‐6, and soluble adhesion molecules as predictors of progressive peripheral atherosclerosis in the general population: Edinburgh artery study. Circulation. 2005;112(7):976‐983. 16087797 10.1161/CIRCULATIONAHA.104.513085

[31] Vainas T , Stassen FRM , De Graaf R , et al. C‐reactive protein in peripheral arterial disease: relation to severity of the disease and to future cardiovascular events. J Vasc Surg. 2005;42(2):243‐251. 16102622 10.1016/j.jvs.2005.03.060

[32] Aboyans V , Criqui MH , Denenberg JO , Knoke JD , Ridker PM , Fronek A . Risk factors for progression of peripheral arterial disease in large and small vessels. Circulation. 2006;113(22):2623‐2629. 16735675 10.1161/CIRCULATIONAHA.105.608679

[33] Musicant SE , Taylor LM , Peters D , et al. Prospective evaluation of the relationship between C‐reactive protein, D‐dimer and progression of peripheral arterial disease. J Vasc Surg. 2006;43(4):772‐780. 16616235 10.1016/j.jvs.2005.12.051

[34] McDermott MM , Liu K , Guralnik JM , et al. Functional decline in patients with and without peripheral arterial disease: predictive value of annual changes in levels of C‐reactive protein and D‐dimer. J Gerontol A Biol Sci Med Sci. 2006;61(4):374‐379. 16611704 10.1093/gerona/61.4.374

[35] Urbonaviciene G , Frystyk J , Flyvbjerg A , Urbonavicius S , Henneberg EW , Lindholt JS . Markers of inflammation in relation to long‐term cardiovascular mortality in patients with lower‐extremity peripheral arterial disease. Int J Cardiol. 2012;160(2):89‐94. 21463908 10.1016/j.ijcard.2011.03.030

[36] Fukase T , Dohi T , Kato Y , et al. Long‐term impact of high‐sensitivity C‐reactive protein in patients with intermittent claudication due to peripheral artery disease following endovascular treatment. Heart Vessel. 2021;36(11):1670‐1678. 10.1007/s00380-021-01863-6 33956183

[37] Gremmels H , Teraa M , de Jager SCA , Pasterkamp G , de Borst GJ , Verhaar MC . A pro‐inflammatory biomarker‐profile predicts amputation‐free survival in patients with severe limb ischemia. Sci Rep. 2019;9(1):10740. 31341203 10.1038/s41598-019-47217-1 PMC6656730

[38] Hogh AL , Joensen J , Lindholt JS , Jacobsen MR , Ostergaard L . C‐reactive protein predicts future arterial and cardiovascular events in patients with symptomatic peripheral arterial disease. Vasc Endovasc Surg. 2008;42(4):341‐347. 10.1177/1538574408316138 18458051

[39] Vidula H , Tian L , Liu K , et al. Biomarkers of inflammation and thrombosis as predictors of near‐term mortality in patients with peripheral arterial disease: a cohort study. Ann Intern Med. 2008;148(2):85‐93. 18195333 10.7326/0003-4819-148-2-200801150-00003 PMC2653260

[40] Criqui MH , Ho LA , Denenberg JO , Ridker PM , Wassel CL , McDermott MM . Biomarkers in peripheral arterial disease patients and near‐ and longer‐term mortality. J Vasc Surg. 2010;52(1):85‐90. 20471776 10.1016/j.jvs.2010.02.004 PMC4077155

[41] Brevetti G , Schiano V , Laurenzano E , et al. Myeloperoxidase, but not C‐reactive protein, predicts cardiovascular risk in peripheral arterial disease. Eur Heart J. 2008;29(2):224‐230. 18156137 10.1093/eurheartj/ehm587

[42] McDermott MMG , Liu K , Green D , et al. Changes in D‐dimer and inflammatory biomarkers before ischemic events in patients with peripheral artery disease: the BRAVO study. Vasc Med. 2016;21(1):12‐20. 26647446 10.1177/1358863X15617541 PMC6615466

[43] Singh TP , Morris DR , Smith S , Moxon JV , Golledge J . Systematic review and meta‐analysis of the association between C‐reactive protein and major cardiovascular events in patients with peripheral artery disease. Eur J Vasc Endovasc Surg. 2017;54:220‐233. 28666785 10.1016/j.ejvs.2017.05.009

[44] Doweik L , Maca T , Schillinger M , Budinsky A , Sabeti S , Minar E . Fibrinogen predicts mortality in high risk patients with peripheral artery disease. Eur J Vasc Endovasc Surg. 2003;26(4):381‐386. 14511999 10.1016/s1078-5884(03)00340-x

[45] Bartlett JW , De Stavola BL , Meade TW . Assessing the contribution of fibrinogen in predicting risk of death in men with peripheral arterial disease. J Thromb Haemost. 2009;7(2):270‐276. 19036067 10.1111/j.1538-7836.2008.03236.x

[46] Altes P , Perez P , Esteban C , et al. Raised fibrinogen levels and outcome in outpatients with peripheral artery disease. Angiology. 2018;69(6):507‐512. 29113452 10.1177/0003319717739720

[47] Kremers B , Wübbeke L , Mees B , Ten Cate H , Spronk H , Ten Cate‐Hoek A . Plasma biomarkers to predict cardiovascular outcome in patients with peripheral artery disease: a systematic review and meta‐analysis. Arterioscler Thromb Vasc Biol. 2020;40:2018‐2032. 32640905 10.1161/ATVBAHA.120.314774 PMC7447177

[48] Silvestro A , Brevetti G , Shiano V , Scopacasa F , Chiariello M . Adhesion molecules and cardiovascular risk in peripheral arterial disease. Soluble vascular cell adhesion molecule‐I improves risk stratification. Thromb Haemost. 2005;93(3):559‐563. 15735810 10.1160/TH04-07-0440

[49] Folsom AR , Pankow JS , Tracy RP , Arnett DK , Peacock JM , Hong Y . Association of C‐reactive protein atherosclerotic disease. J Cardiol. 2001;88(1):112‐117. 10.1016/s0002-9149(01)01603-4 11448405

[50] Cassar K , Bachoo P , Ford I , Greaves M , Brittenden J . Markers of coagulation activation, endothelial stimulation and inflammation in patients with peripheral arterial disease. Eur J Vasc Endovasc Surg. 2005;29(2):171‐176. 15649725 10.1016/j.ejvs.2004.11.001

[51] Edlinger C , Lichtenauer M , Wernly B , et al. Disease‐specific characteristics of vascular cell adhesion molecule‐1 levels in patients with peripheral artery disease. Heart Vessel. 2019;34(6):976‐983. 10.1007/s00380-018-1315-1 PMC6531410 30535754

[52] McDermott MMG , Greenland P , Green D , et al. D‐dimer, inflammatory markers, and lower extremity functioning in patients with and without peripheral arterial disease. Circulation. 2003;107(25):3191‐3198. 12810614 10.1161/01.CIR.0000074227.53616.CC

[53] McDermott MM , Liu K , Ferrucci L , et al. Circulating blood markers and functional impairment in peripheral arterial disease. J Am Geriatr Soc. 2008;56(8):1504‐1510. 18662216 10.1111/j.1532-5415.2008.01797.x PMC2658758

[54] Nylænde M , Kroese A , Stranden E , et al. Markers of vascular inflammation are associated with the extent of atherosclerosis assessed as angiographic score and treadmill walking distances in patients with peripheral arterial occlusive disease. Vasc Med. 2006;11(1):21‐28. 16669409 10.1191/1358863x06vm662oa

[55] McDermott MM , Liu K , Ferrucci L , et al. Relation of interleukin‐6 and vascular cellular adhesion molecule‐1 levels to functional decline in patients with lower extremity peripheral arterial disease. Am J Cardiol. 2011;107(9):1392‐1398. 21371679 10.1016/j.amjcard.2011.01.007 PMC3227858

[56] McDermott MMG , Guralnik JM , Corsi A , et al. Patterns of inflammation associated with peripheral arterial disease: the InCHIANTI study. Am Heart J. 2005;150(2):276‐281. 16086930 10.1016/j.ahj.2004.09.032

[57] Wildman RP , Muntner P , Chen J , Sutton‐Tyrrell K , He J . Relation of inflammation to peripheral arterial disease in the National Health and nutrition examination survey, 1999‐2002. Am J Cardiol. 2005;96(11):1579‐1583. 16310445 10.1016/j.amjcard.2005.07.067

[58] Murabito JM , Keyes MJ , Guo CY , et al. Cross‐sectional relations of multiple inflammatory biomarkers to peripheral arterial disease: the Framingham offspring study. Atherosclerosis. 2009;203(2):509‐514. 18701106 10.1016/j.atherosclerosis.2008.06.031 PMC2690511

[59] Cauley JA , Kassem AM , Lane NE , Thorson S . Prevalent peripheral arterial disease and inflammatory burden. BMC Geriatr. 2016;16(1):1‐9. 27938334 10.1186/s12877-016-0389-9 PMC5148838

[60] Signorelli SS , Anzaldi M , Libra M , et al. Plasma levels of inflammatory biomarkers in peripheral arterial disease: results of a cohort study. Angiology. 2016;67(9):870‐874. 26888895 10.1177/0003319716633339

[61] Vyas MV , Mrkobrada M , Donner A , Hackam DG . Underrepresentation of peripheral artery disease in modern cardiovascular trials: systematic review and meta‐analysis. Int J Cardiol. 2013;168(5):4875‐4876. 23896535 10.1016/j.ijcard.2013.07.050

[62] Yeh ST , Morton DJ , Barrett‐Connor E . Lower extremity arterial disease in older women: the rancho Bernardo study. J Women Health Gend Based Med. 2000;9(4):373‐380. 10.1089/15246090050020682 10868609

[63] Kannel WB , McGee DL . Update on some epidemiologic features of intermittent claudication: the Framingham study. J Am Geriatr Soc. 1985;33(1):13‐18. 3965550 10.1111/j.1532-5415.1985.tb02853.x

[64] Brevetti G , Bucur R , Balbarini A , et al. Women and peripheral arterial disease: same disease, different issues. J Cardiovasc Med. 2008;9(4):382‐388. 10.2459/JCM.0b013e3282f03b90 18334893

[65] Haine A , Kavanagh S , Berger JS , et al. Sex‐specific risks of major cardiovascular and limb events in patients with symptomatic peripheral artery disease. J Am Coll Cardiol. 2020;75(6):608‐617. 32057375 10.1016/j.jacc.2019.11.057

[66] Chase‐Vilchez AZ , Chan IHY , Peters SAE , Woodward M . Diabetes as a risk factor for incident peripheral arterial disease in women compared to men: a systematic review and meta‐analysis. Cardiovasc Diabetol. 2020;19:151. 32979922 10.1186/s12933-020-01130-4 PMC7520021

[67] Vouyouka AG , Egorova NN , Salloum A , et al. Lessons learned from the analysis of gender effect on risk factors and procedural outcomes of lower extremity arterial disease. J Vasc Surg. 2010;52(5):1196‐1202. 20674247 10.1016/j.jvs.2010.05.106

[68] Xu Y , Pouncey AL , Zhou Z , Woodward M , Harris K . Smoking as a risk factor for lower extremity peripheral artery disease in women compared to men: a systematic review and meta‐analysis. PLoS One. 2024;19:e0300963. 38656947 10.1371/journal.pone.0300963 PMC11042699

[69] Xu Y , Harris K , Pouncey AL , et al. Sex differences in risk factors for incident peripheral artery disease hospitalisation or death: Cohort study of UK Biobank participants. PLoS One. 2023;18:e0292083. 37851596 10.1371/journal.pone.0292083 PMC10584119

[70] Wang J , Ruotsalainen S , Moilanen L , Lepistö P , Laakso M , Kuusisto J . Metabolic syndrome and incident end‐stage peripheral vascular disease: a 14‐year follow‐up study in elderly Finns. Diabetes Care. 2007;30(12):3099‐3104. 17848614 10.2337/dc07-0985

[71] Chen Q , Zhu H , Shen F , et al. Sex‐influenced association of metabolic syndrome with lower extremity arterial disease in type 2 diabetes. J Diabetes Complicat. 2020;34(5):107537. 10.1016/j.jdiacomp.2020.107537 32107122

[72] Conen D , Rexrode KM , Creager MA , Ridker PM , Pradhan AD . Metabolic syndrome, inflammation, and risk of symptomatic peripheral artery disease in women: a prospective study. Circulation. 2009;120(12):1041‐1047. 19738135 10.1161/CIRCULATIONAHA.109.863092 PMC2763563

[73] Choi J , Joseph L , Pilote L . Obesity and C‐reactive protein in various populations: a systematic review and meta‐analysis. Obes Rev. 2013;14(3):232‐244. 23171381 10.1111/obr.12003

[74] Lakoski SG , Cushman M , Criqui M , et al. Gender and C‐reactive protein: data from the multiethnic study of atherosclerosis (MESA) cohort. Am Heart J. 2006;152(3):593‐598. 16923436 10.1016/j.ahj.2006.02.015

[75] Srivaratharajah K , Abramson BL . Women and peripheral arterial disease: a review of sex differences in epidemiology, clinical manifestations, and outcomes. Can J Cardiol. 2018;34(4):356‐361. 29571419 10.1016/j.cjca.2018.01.009

[76] Wang GJ , Shaw PA , Townsend RR , et al. Sex differences in the incidence of peripheral artery disease in the chronic renal insufficiency cohort. Circ Cardiovasc Qual Outcomes. 2016;9(2_suppl_1):S86‐S93. 26908866 10.1161/CIRCOUTCOMES.115.002180 PMC4770580

[77] Higgins JP , Higgins JA . Epidemiology of peripheral arterial disease in women. J Epidemiol. 2003;13(1):1‐14. 12587608 10.2188/jea.13.1 PMC9538614

[78] Diehm C , Schuster A , Allenberg JR , et al. High prevalence of peripheral arterial disease and co‐morbidity in 6880 primary care patients: cross‐sectional study. Atherosclerosis. 2004;172(1):95‐105. 14709362 10.1016/s0021-9150(03)00204-1

[79] Hirsch AT , Allison MA , Gomes AS , et al. A call to action: women and peripheral artery disease: a scientific statement from the american heart association. Circulation. 2012;125(11):1449‐1472. 22343782 10.1161/CIR.0b013e31824c39ba

[80] Sigvant B , Wiberg‐Hedman K , Bergqvist D , et al. A population‐based study of peripheral arterial disease prevalence with special focus on critical limb ischemia and sex differences. J Vasc Surg. 2007;45(6):1185‐1191. 17543683 10.1016/j.jvs.2007.02.004

[81] Hiramoto JS , Katz R , Weisman S , Conte M . Gender‐specific risk factors for peripheral artery disease in a voluntary screening population. J Am Heart Assoc. 2014;3(2):1‐9. 10.1161/JAHA.113.000651 PMC4187488 24627420

[82] Song P , Rudan D , Zhu Y , et al. Global, regional, and national prevalence and risk factors for peripheral artery disease in 2015: an updated systematic review and analysis. Lancet Glob Health. 2019;7(8):e1020‐e1030. 31303293 10.1016/S2214-109X(19)30255-4

[83] Maggio M , Cattabiani C , Lauretani F , et al. The relationship between sex hormones, sex hormone binding globulin and peripheral artery disease in older persons. Atherosclerosis. 2012;225(2):469‐474. 23102785 10.1016/j.atherosclerosis.2012.09.014 PMC4050374

[84] Ryczkowska K , Adach W , Janikowski K , Banach M , Bielecka‐Dabrowa A . Menopause and women's cardiovascular health: is it really an obvious relationship? Arch Med Sci. 2023;19(2):458‐466. 37034510 10.5114/aoms/157308 PMC10074318

[85] Song DK , Sung YA , Hong YS , Kim MH , Lee H . Impact of sex and menopausal hormonal therapy on cardiovascular diseases in people with diabetes or prediabetes. Sci Rep. 2025;15(1):25450. 40659706 10.1038/s41598-025-01768-8 PMC12259954

[86] Hsia J , Criqui MH , Rodabough RJ , et al. Estrogen plus progestin and the risk of peripheral arterial disease: the Women's Health Initiative. Circulation. 2004;109(5):620‐626. 14769684 10.1161/01.CIR.0000115309.63979.92

[87] Hulley S , Grady D , Bush T , et al. Randomized trial of estrogen plus progestin for secondary prevention of coronary heart disease in postmenopausal women. Heart and estrogen/progestin replacement study (HERS) research group. JAMA. 1998;280(7):605‐613. 9718051 10.1001/jama.280.7.605

[88] O'Hare AM , Vittinghoff E , Hsia J , Shlipak MG . Renal insufficiency and the risk of lower extremity peripheral arterial disease: results from the heart and estrogen/progestin replacement study (HERS). J Am Soc Nephrol. 2004;15(4):1046‐1051. 15034108 10.1097/01.asn.0000119574.27772.fd

[89] McDermott MMG , Greenland P , Liu K , et al. Sex differences in peripheral arterial disease: leg symptoms and physical functioning. J Am Geriatr Soc. 2003;51(2):222‐228. 12558719 10.1046/j.1532-5415.2003.51061.x

[90] Porras CP , Bots ML , Teraa M , van Doorn S , Vernooij RWM . Differences in symptom presentation in women and men with confirmed lower limb peripheral artery disease: a systematic review and meta‐analysis. Eur J Vasc Endovasc Surg. 2022;63:602‐612. 35248439 10.1016/j.ejvs.2021.12.039

[91] McDermott MM , Ferrucci L , Liu K , et al. Women with peripheral arterial disease experience faster functional decline than men with peripheral arterial disease. J Am Coll Cardiol. 2011;57(6):707‐714. 21292130 10.1016/j.jacc.2010.09.042 PMC5077144

[92] Diehm N , Shang A , Silvestro A , et al. Association of cardiovascular risk factors with pattern of lower limb atherosclerosis in 2659 patients undergoing angioplasty. Eur J Vasc Endovasc Surg. 2006;31(1):59‐63. 16269257 10.1016/j.ejvs.2005.09.006

[93] Ortmann J , Nüesch E , Traupe T , Diehm N , Baumgartner I . Gender is an independent risk factor for distribution pattern and lesion morphology in chronic critical limb ischemia. J Vasc Surg. 2012;55(1):98‐104. 22112554 10.1016/j.jvs.2011.07.074

[94] Gardner AW , Parker DE , Montgomery PS , Blevins SM . Diabetic women are poor responders to exercise rehabilitation in the treatment of claudication. J Vasc Surg. 2014;59(4):1036‐1043. 24246541 10.1016/j.jvs.2013.10.058 PMC3966945

[95] Bloemenkamp DGM , Van Den Bosch MAAJ , Mali WPTM , et al. Novel risk factors for peripheral arterial disease in young women. Am J Med. 2002;113(6):462‐467. 12427494 10.1016/s0002-9343(02)01258-5

[96] Pradhan AD , Shrivastava S , Cook NR , Rifai N , Creager MA , Ridker PM . Symptomatic peripheral arterial disease in women: nontraditional biomarkers of elevated risk. Circulation. 2008;117(6):823‐831. 18227386 10.1161/CIRCULATIONAHA.107.719369

[97] Gardner AW , Parker DE , Montgomery PS , et al. Gender and racial differences in endothelial oxidative stress and inflammation in patients with symptomatic peripheral artery disease. J Vasc Surg. 2015;61(5):1249‐1257. 24703977 10.1016/j.jvs.2014.02.045 PMC4185015

[98] Lo RC , Bensley RP , Dahlberg SE , et al. Presentation, treatment, and outcome differences between men and women undergoing revascularization or amputation for lower extremity peripheral arterial disease. J Vasc Surg. 2014;59(2):409‐418. 24080134 10.1016/j.jvs.2013.07.114 PMC3946884

[99] Ferranti KM , Osler TM , Duffy RP , Stanley AC , Bertges DJ . Association between gender and outcomes of lower extremity peripheral vascular interventions. J Vasc Surg. 2015;62(4):990‐997. 26209578 10.1016/j.jvs.2015.03.066

[100] Baubeta Fridh E , Andersson M , Thuresson M , et al. Editor's choice – impact of comorbidity, medication, and gender on amputation rate following revascularisation for chronic limb threatening Ischaemia. Eur J Vasc Endovasc Surg. 2018;56(5):681‐688. 30093176 10.1016/j.ejvs.2018.06.003

[101] De Matteis G , Biscetti F , Della Polla DA , et al. Sex‐based differences in clinical characteristics and outcomes among patients with peripheral artery disease: a retrospective analysis. *Journal of* . Clin Med. 2023;12(15):5094. 10.3390/jcm12155094 PMC10420161 37568498

[102] Mentias A , Vaughan‐Sarrazin M , Saad M , Girotra S . Sex differences in management and outcomes of critical limb ischemia in the Medicare population. Circ Cardiovasc Interv. 2020;13(10):E009459. 33079598 10.1161/CIRCINTERVENTIONS.120.009459 PMC7583656

[103] Wang J , He Y , Shu C , Zhao J , Dubois L . The effect of gender on outcomes after lower extremity revascularization. J Vasc Surg. 2017;65:889‐906.e4. 28236929 10.1016/j.jvs.2016.11.030

[104] Levin MG , Klarin D , Georgakis MK , et al. A missense variant in the IL‐6 receptor and protection from peripheral artery disease. Circ Res. 2021;129(10):968‐970. 34547901 10.1161/CIRCRESAHA.121.319589 PMC8556352

[105] Russell KS , Yates DP , Kramer CM , et al. A randomized, placebo‐controlled trial of canakinumab in patients with peripheral artery disease. Vasc Med. 2019;24(5):414‐421. 31277561 10.1177/1358863X19859072 PMC6791746

